# Supplementary figures and images for: Consequences of platelet-educated cancer cells on the expression of inflammatory and metastatic glycoproteins
Source: PLoS One. 2025 Mar 17;20(3):e0317096. doi: 10.1371/journal.pone.0317096 (PMC11913274; doi:10.1371/journal.pone.0317096)

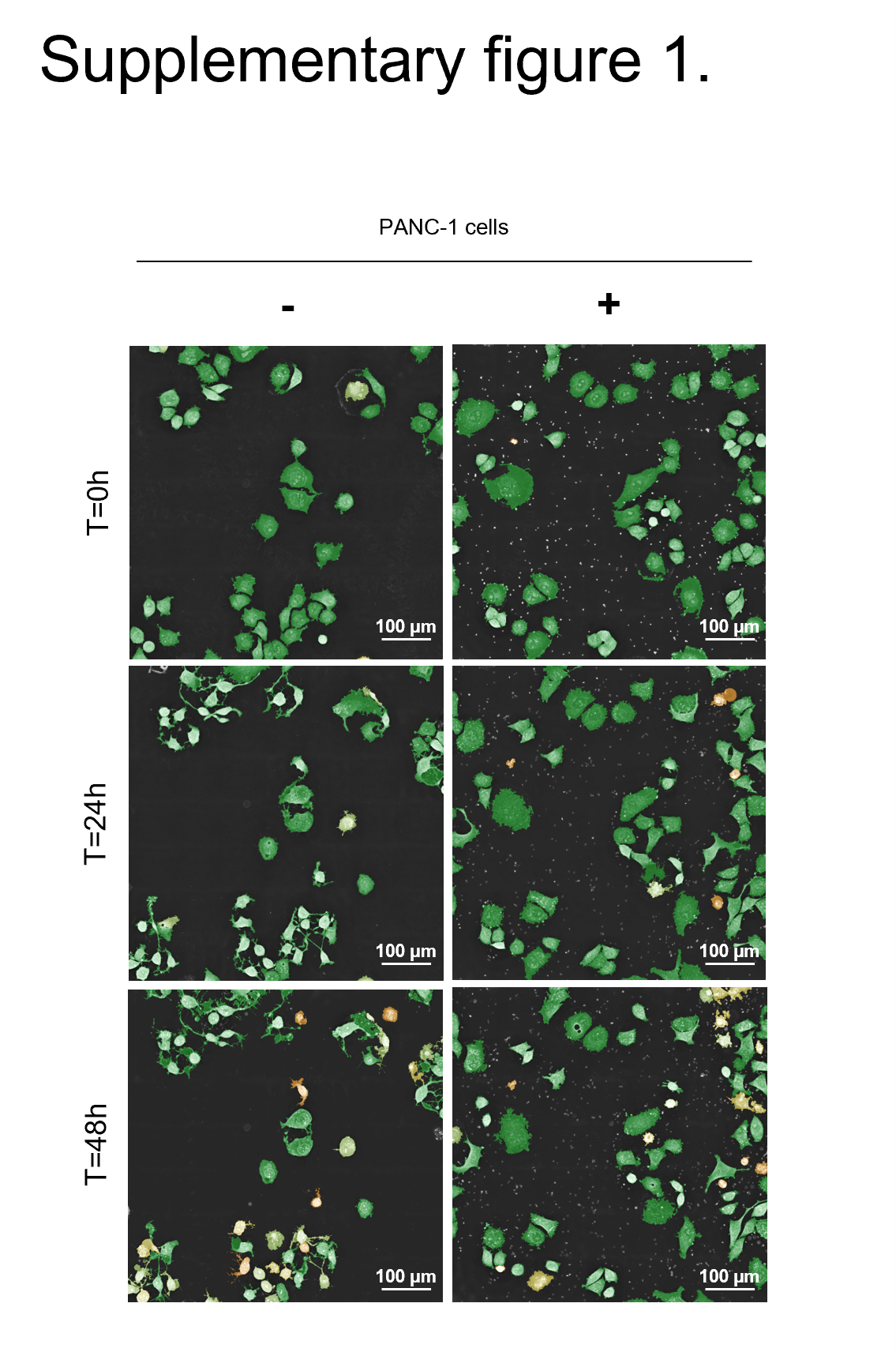

Supplement: S1 Fig — Segmentation was performed using “Cell Death Assay” module, excluding platelets from the analysis. Colors represent live cells (green) or dead cells (yellow). (TIFF) [file pone.0317096.s001.tiff]

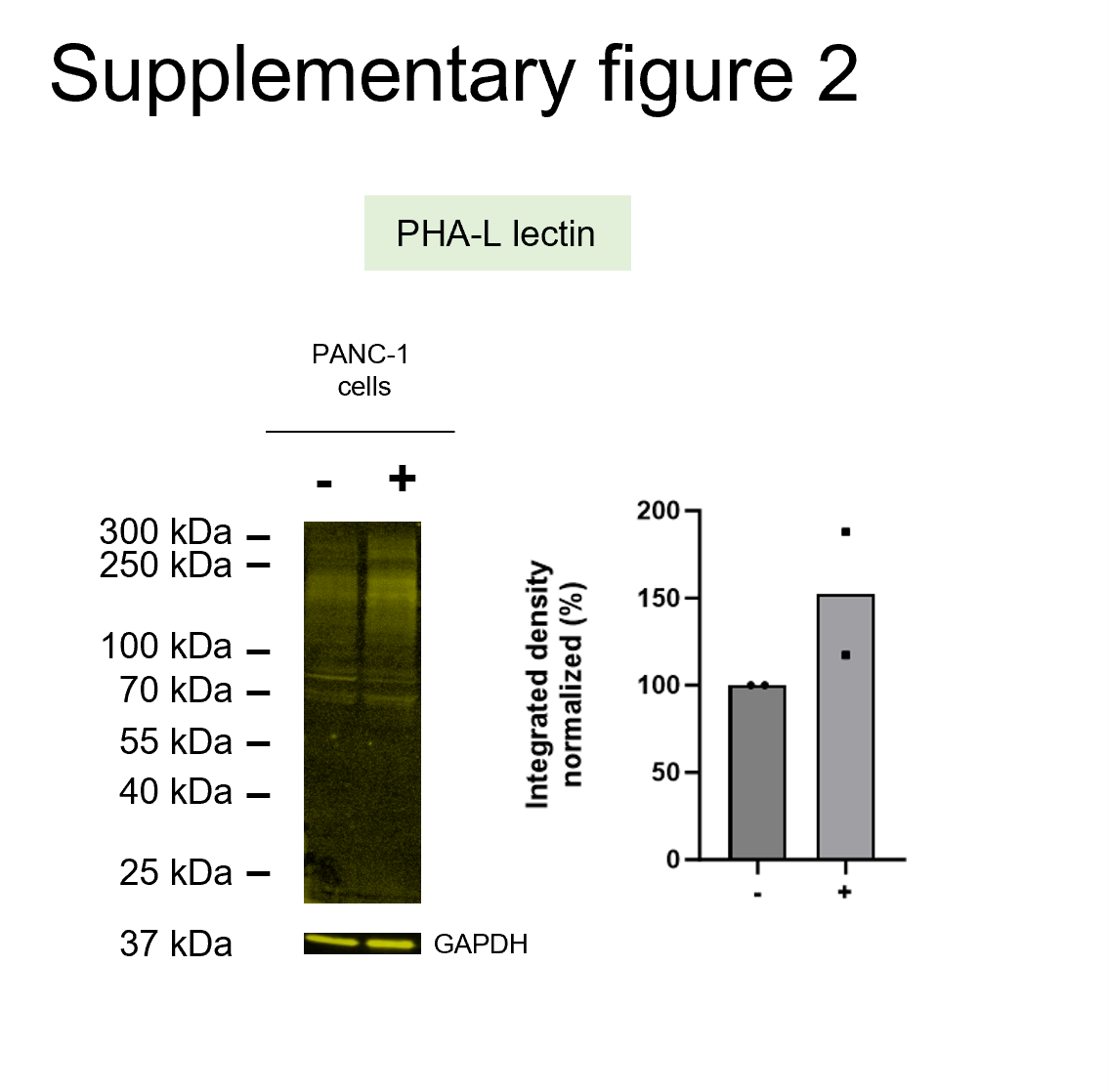

Supplement: S2 Fig — GAPDH expression was detected as a loading control for the experiment. The graphs show the mean of integrated density of glycan motif expression normalized to cancer cells alone. (TIF) [file pone.0317096.s002.tif]

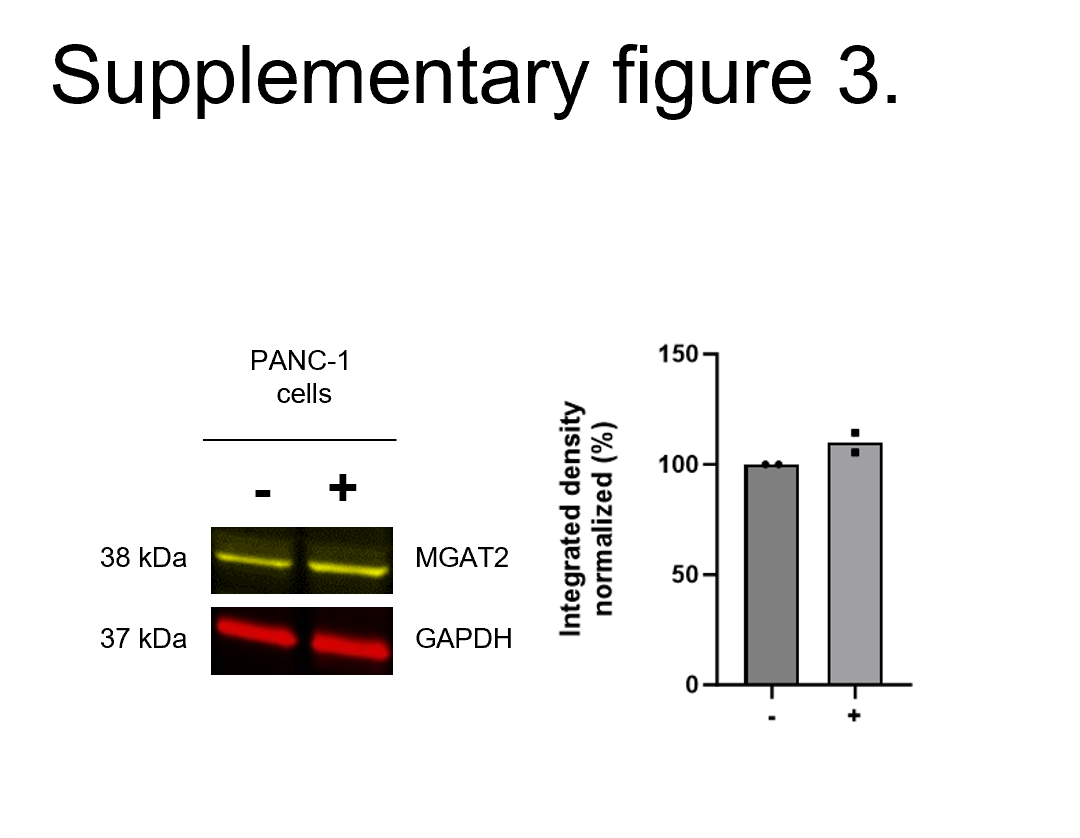

Supplement: S3 Fig — GAPDH expression was detected as a loading control for the experiment. The graphs show the mean of integrated density of MGAT2 expression normalized to cancer cells alone. (TIF) [file pone.0317096.s003.tif]
