# Supplementary material for: Consequences of platelet-educated cancer cells on the expression of inflammatory and metastatic glycoproteins
Source: PLoS One. 2025 Mar 17;20(3):e0317096. doi: 10.1371/journal.pone.0317096 (PMC11913274; doi:10.1371/journal.pone.0317096)

Fig.1E

Western Blot in fluorescence (AF680), exposure time: 673 ms
GALNT3

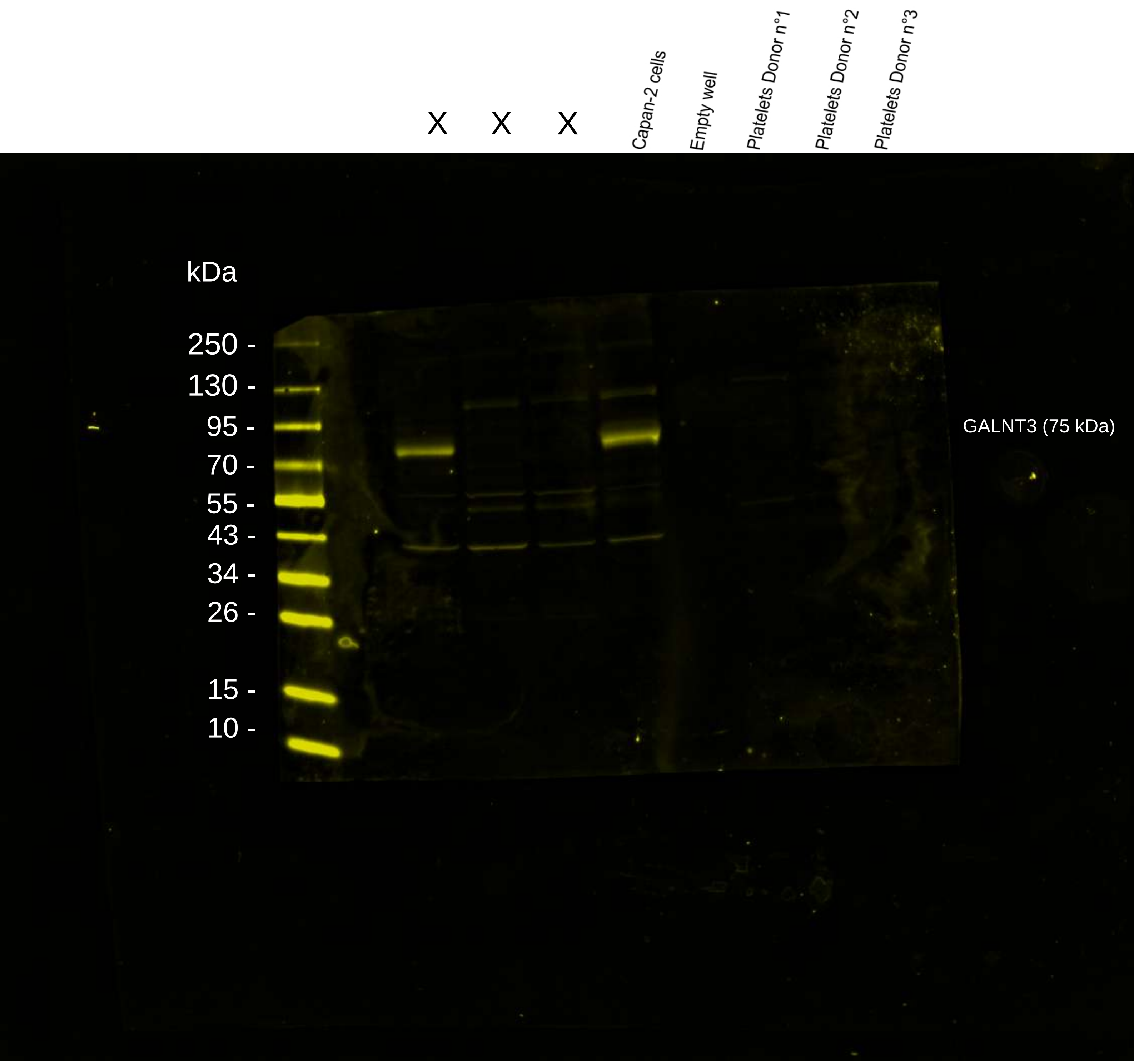

Fig.1E

Western Blot in fluorescence (AF647), exposure time: 1s 518 ms
GAPDH

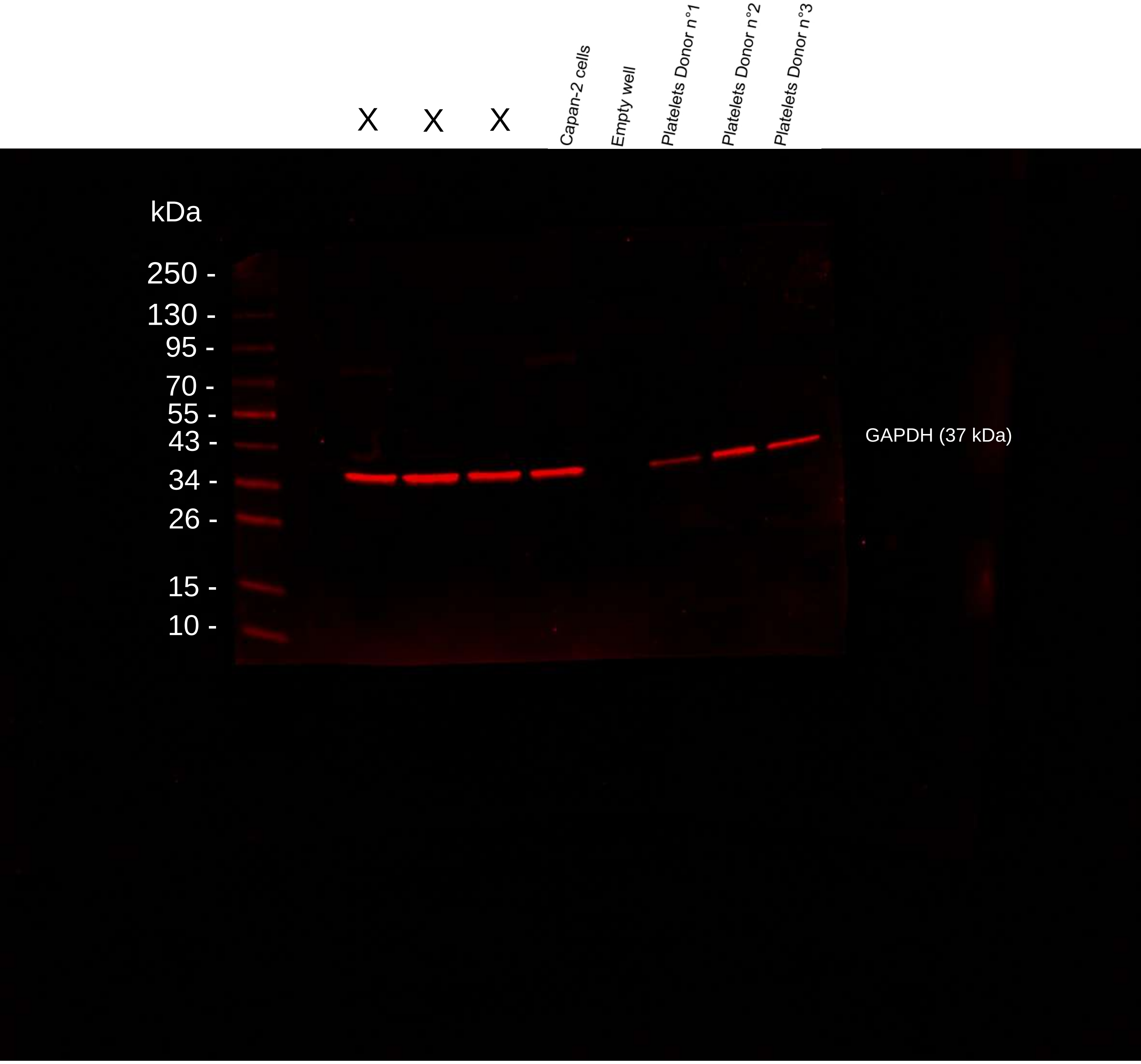

Fig.1G

Western Blot in fluorescence (AF680), time exposure: 10s
GALNT3

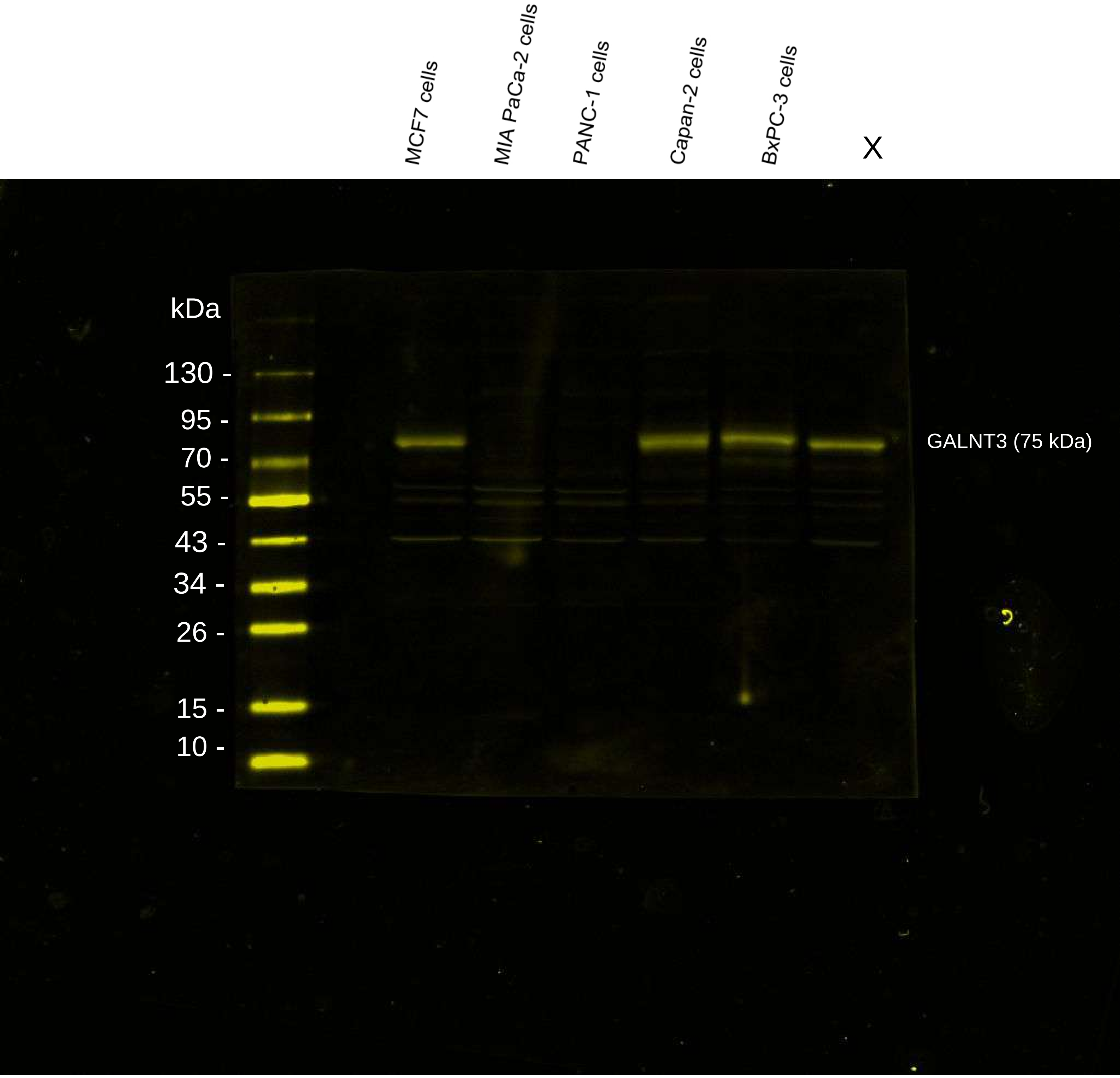

Fig.1G

Western Blot in fluorescence (AF647), time exposure: 1s 987ms
GADPH

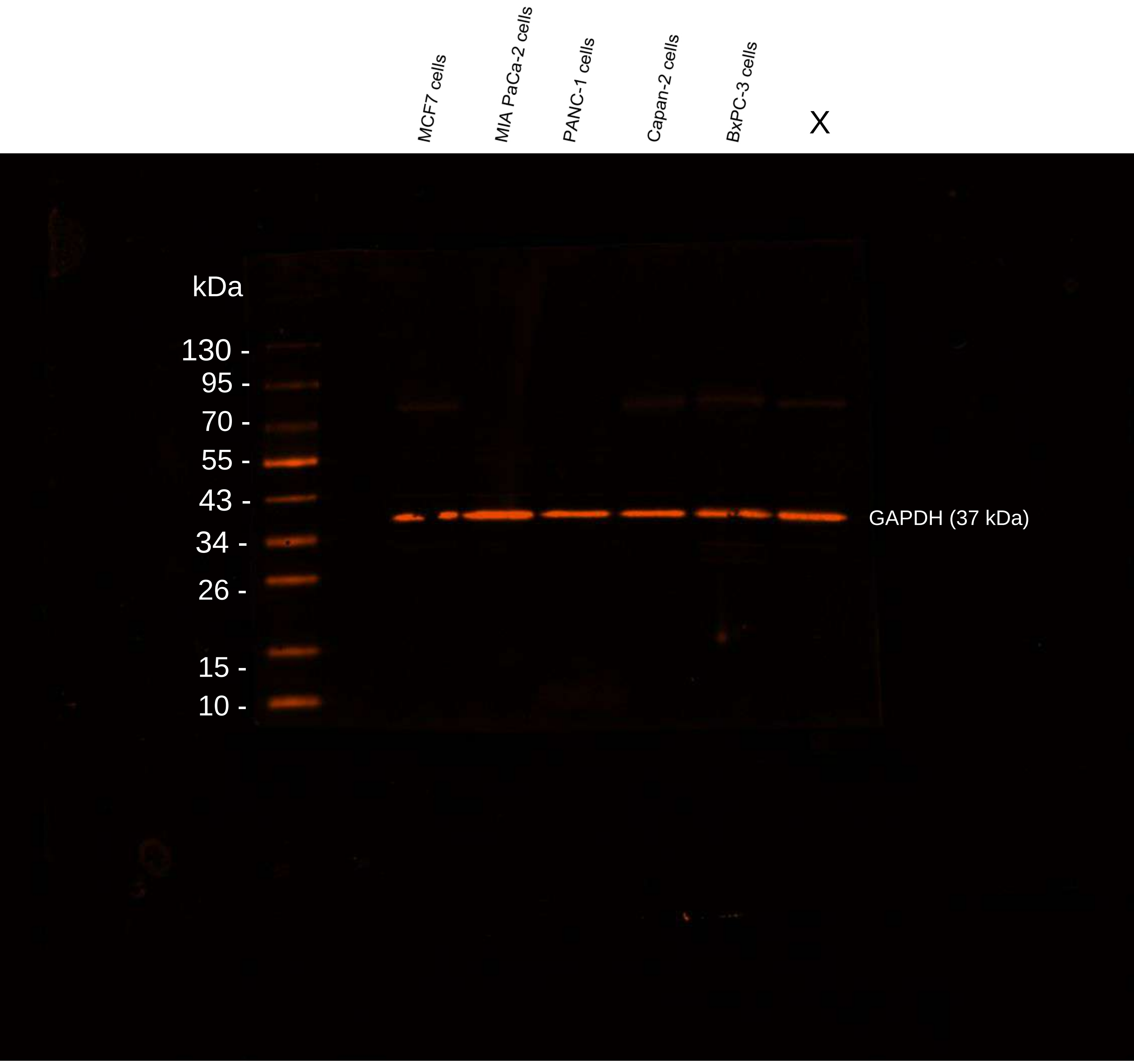

Fig.2B

Western Blot in fluorescence (AF647), exposure time: 3s 540 ms
VVL Lectin (PANC-1)

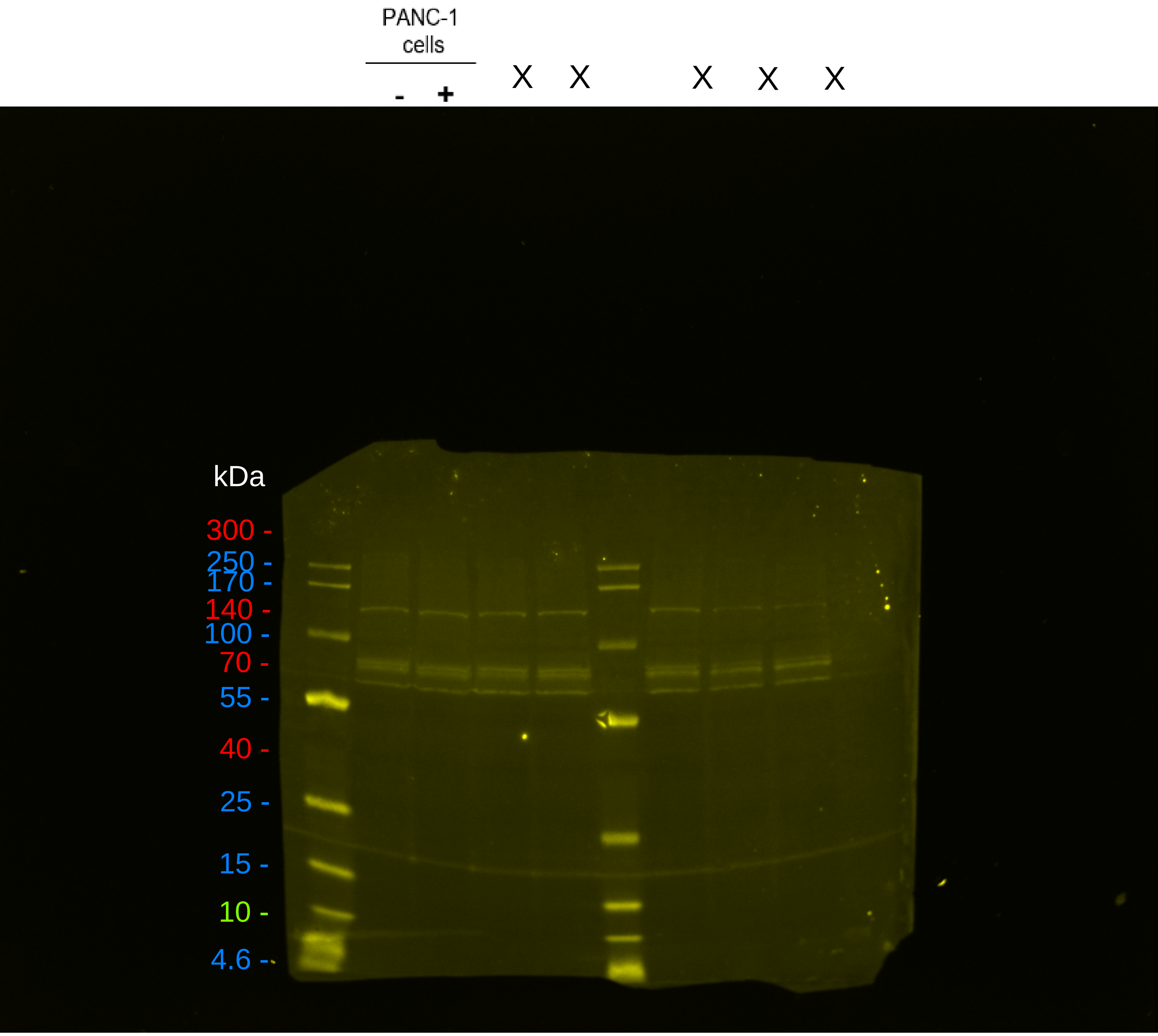

Fig.2B

Western Blot in fluorescence (AF488), exposure time: 545 ms
GAPDH Lectin (PANC-1)

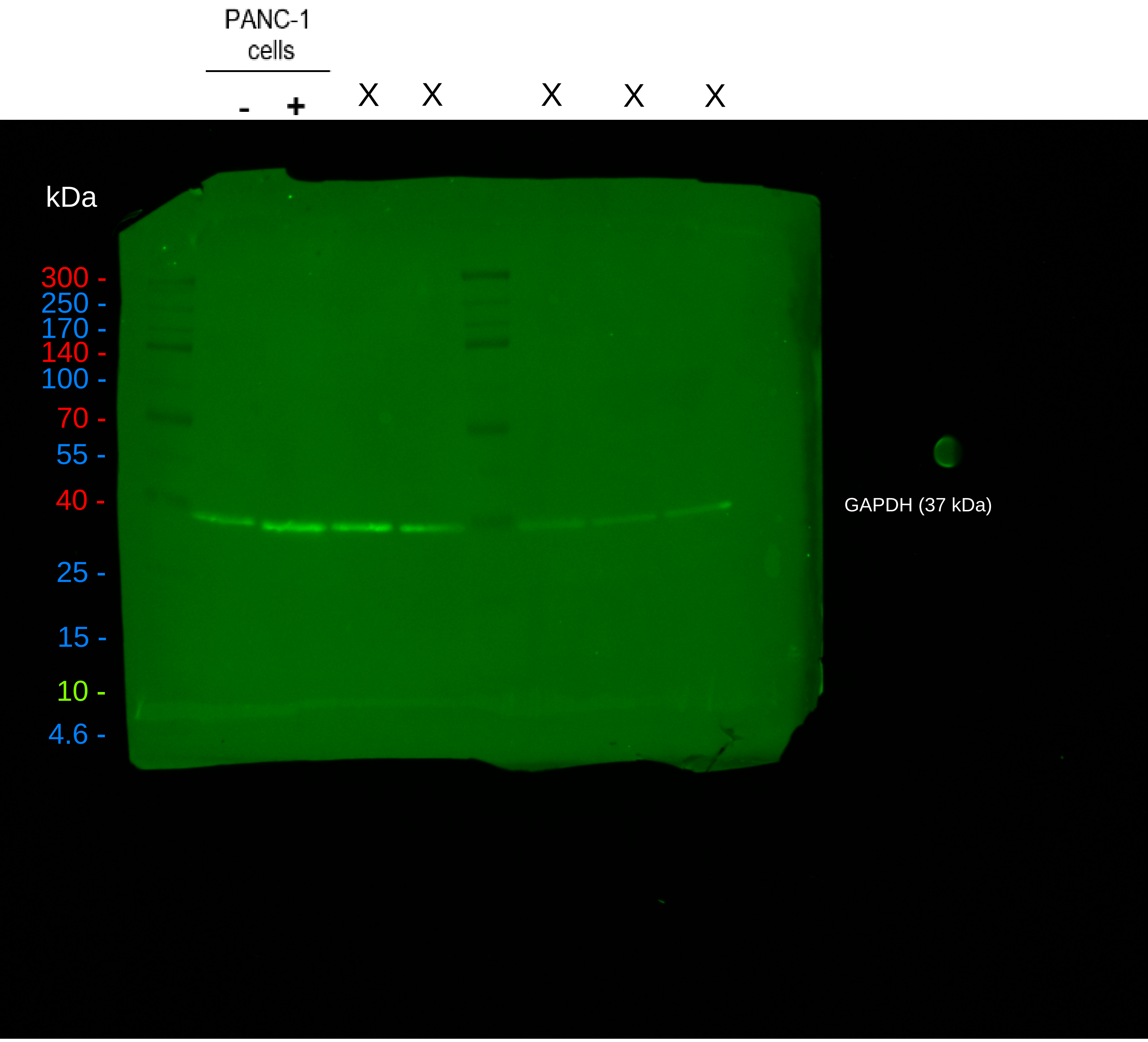

Fig.2B

Western Blot in fluorescence (AF647), exposure time: 3s 632 ms
VVL Lectin (MIA PaCa-2)

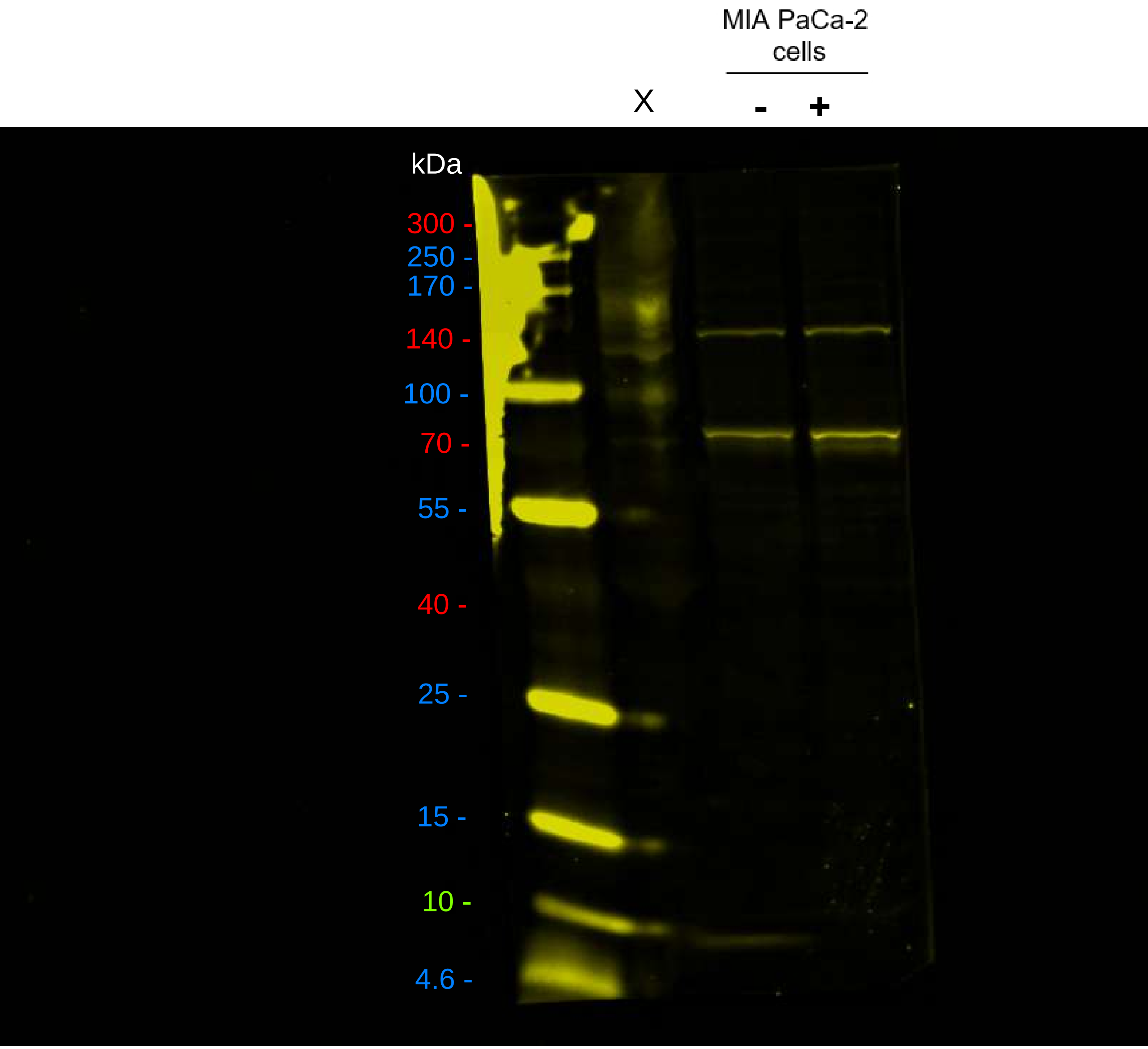

Fig.2B

Western Blot in fluorescence (AF488), multiexposure
GAPDH (MIA PaCa-2)

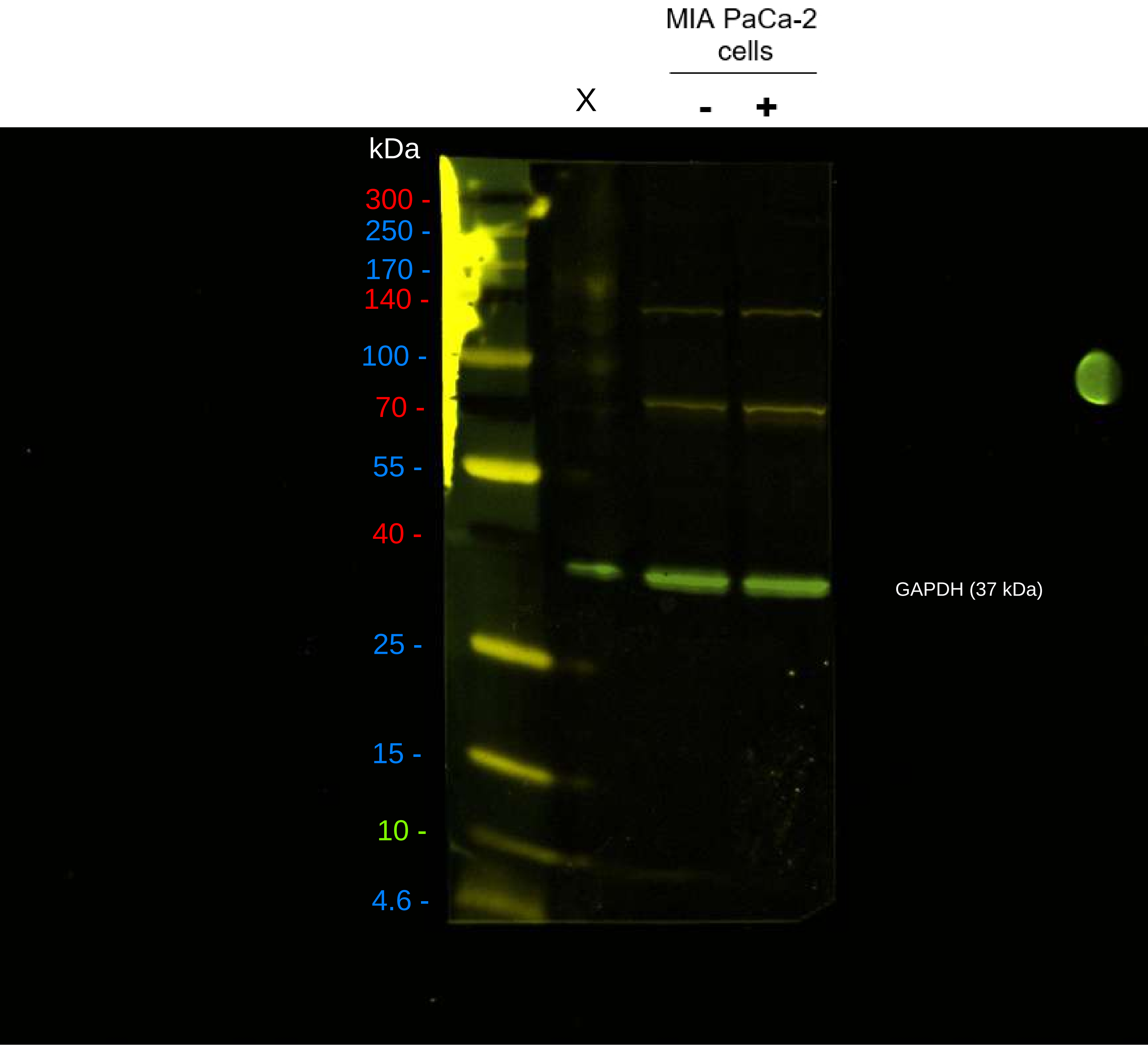

Fig.2B

Western Blot in fluorescence (AF647), exposure time: 5s
VVL Lectin (Capan-2)

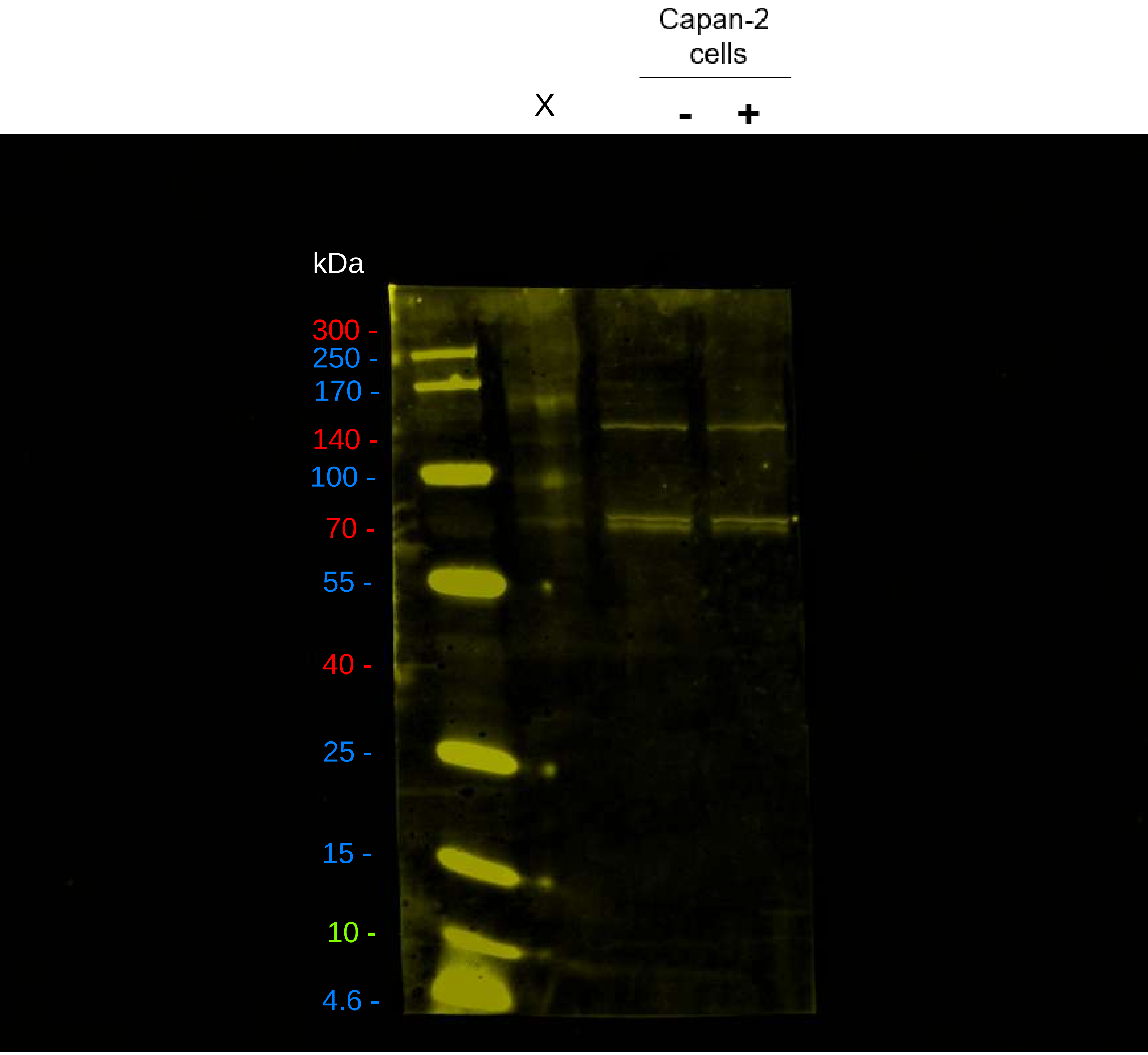

Fig.2B

Western Blot in fluorescence (AF647), exposure time: 2s 790ms
GAPDH (Capan-2)

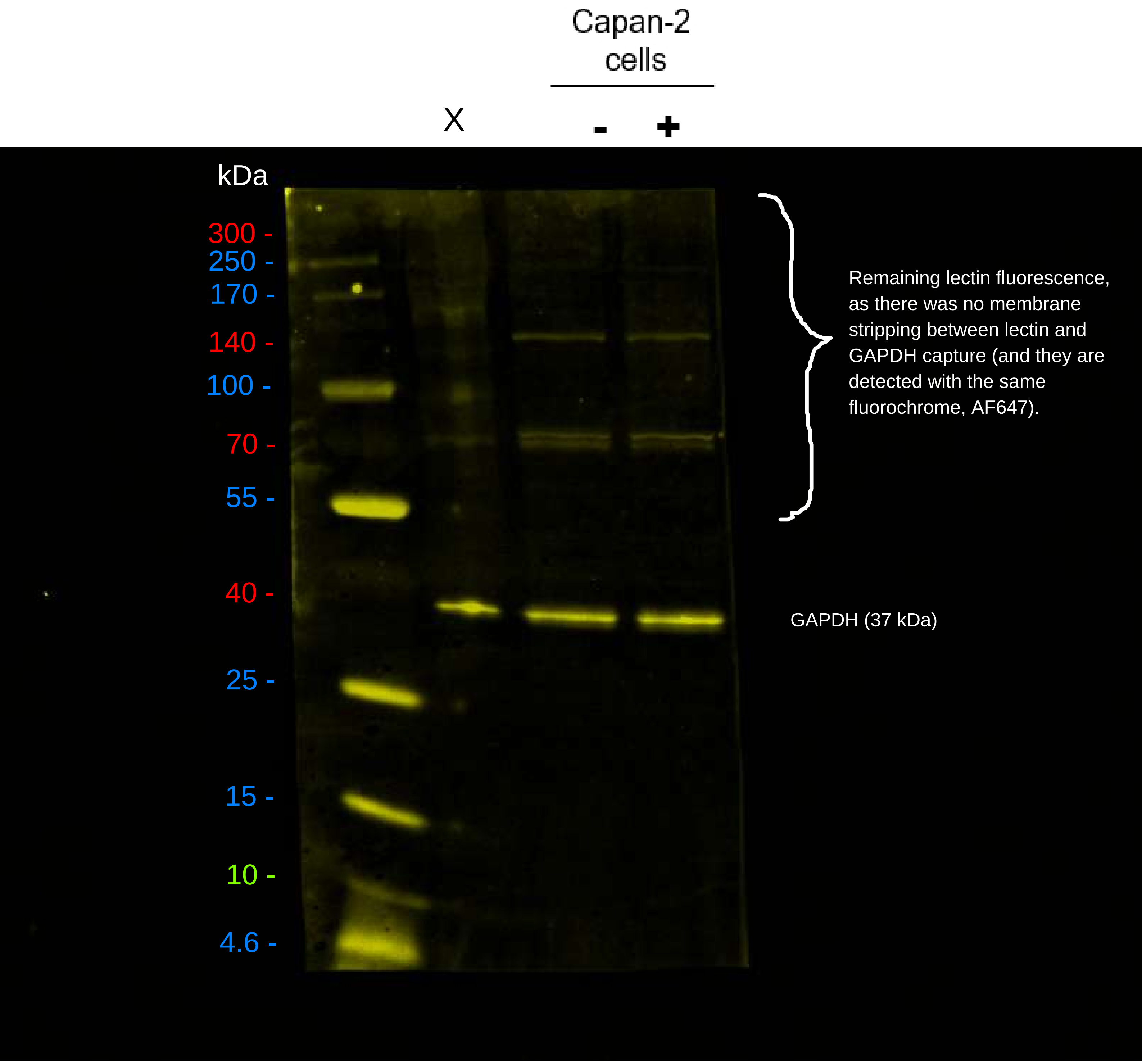

Fig.2B

Western Blot in fluorescence (AF647), exposure time: 218ms
AAL Lectin (PANC-1)

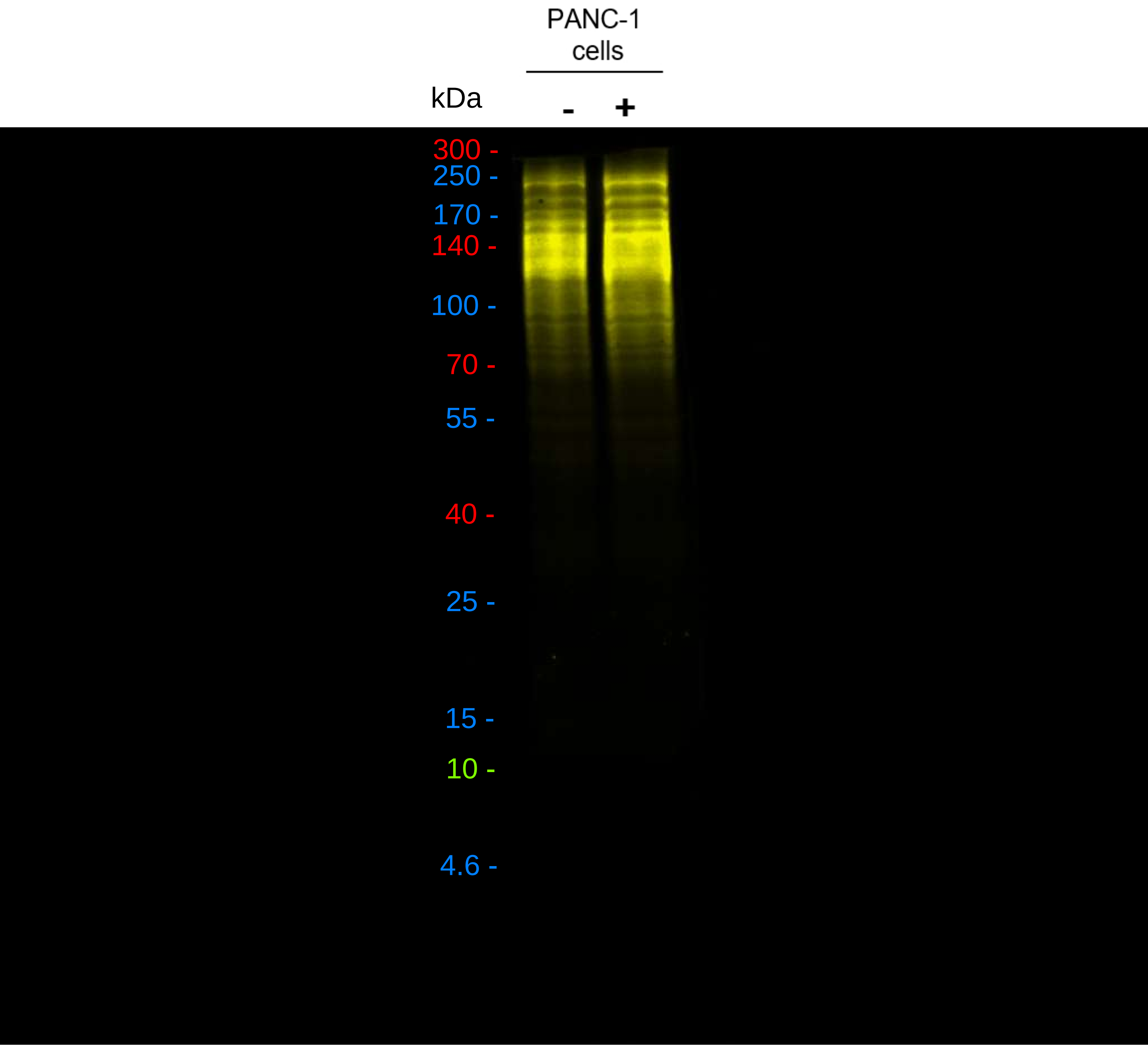

Fig.2B

Western Blot in fluorescence (AF647), multiexposure
GAPDH (PANC-1)

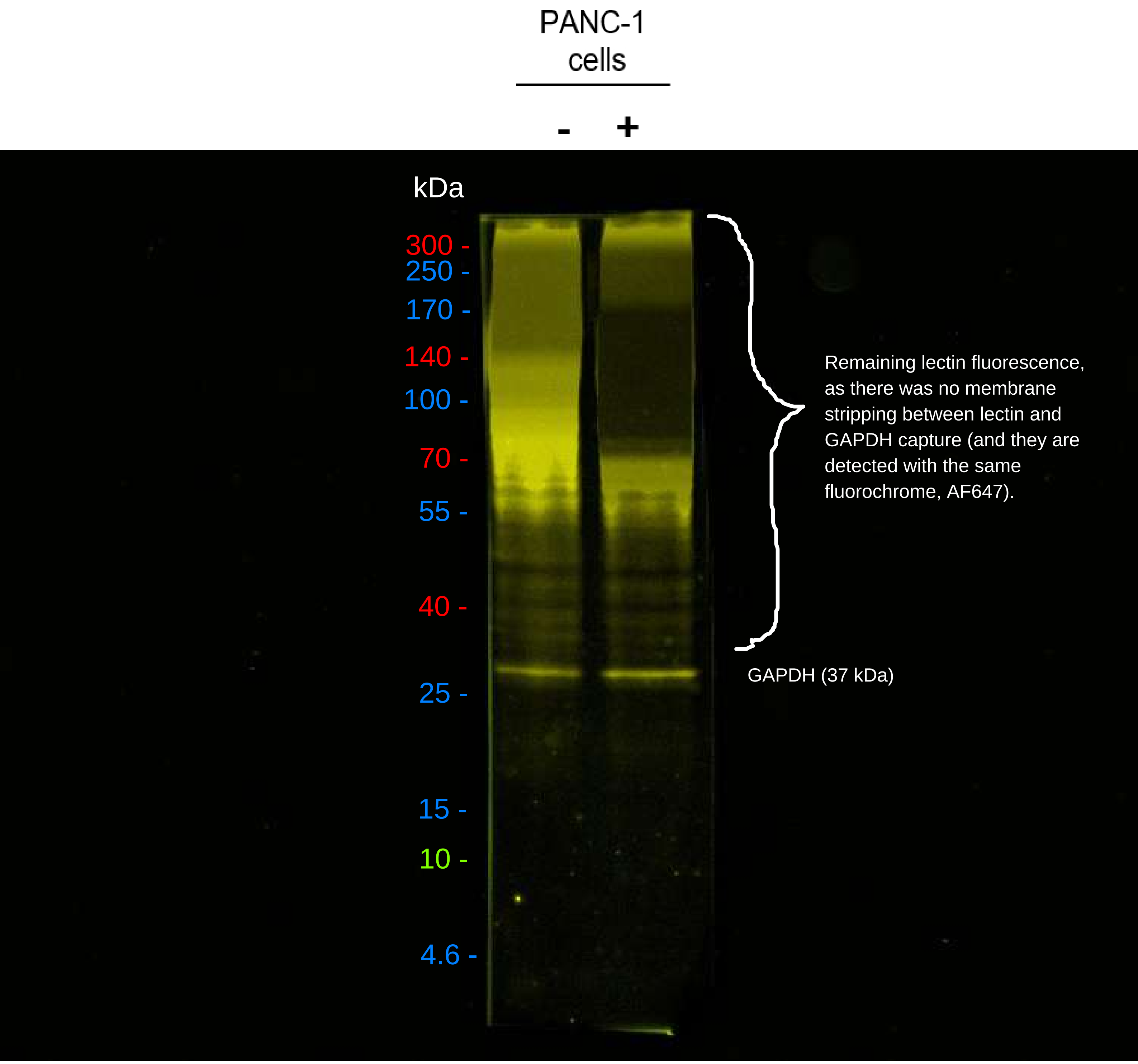

Fig.2B

Western Blot in fluorescence (AF647), exposure time: 353 ms
AAL Lectin (MIA PaCa-2)

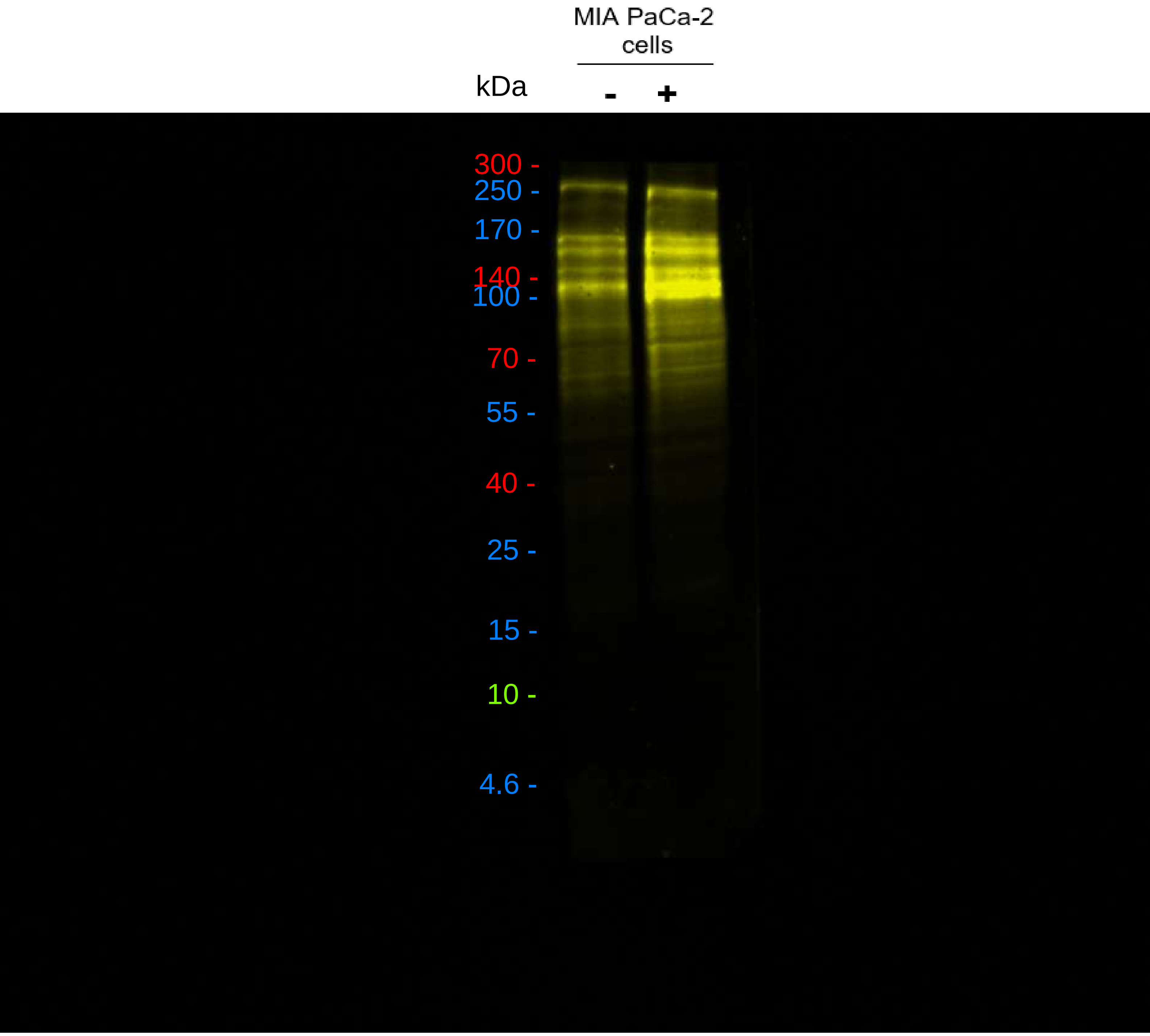

Fig.2B

Western Blot in fluorescence (AF488), multiexposure
GAPDH (MIA PaCa-2)

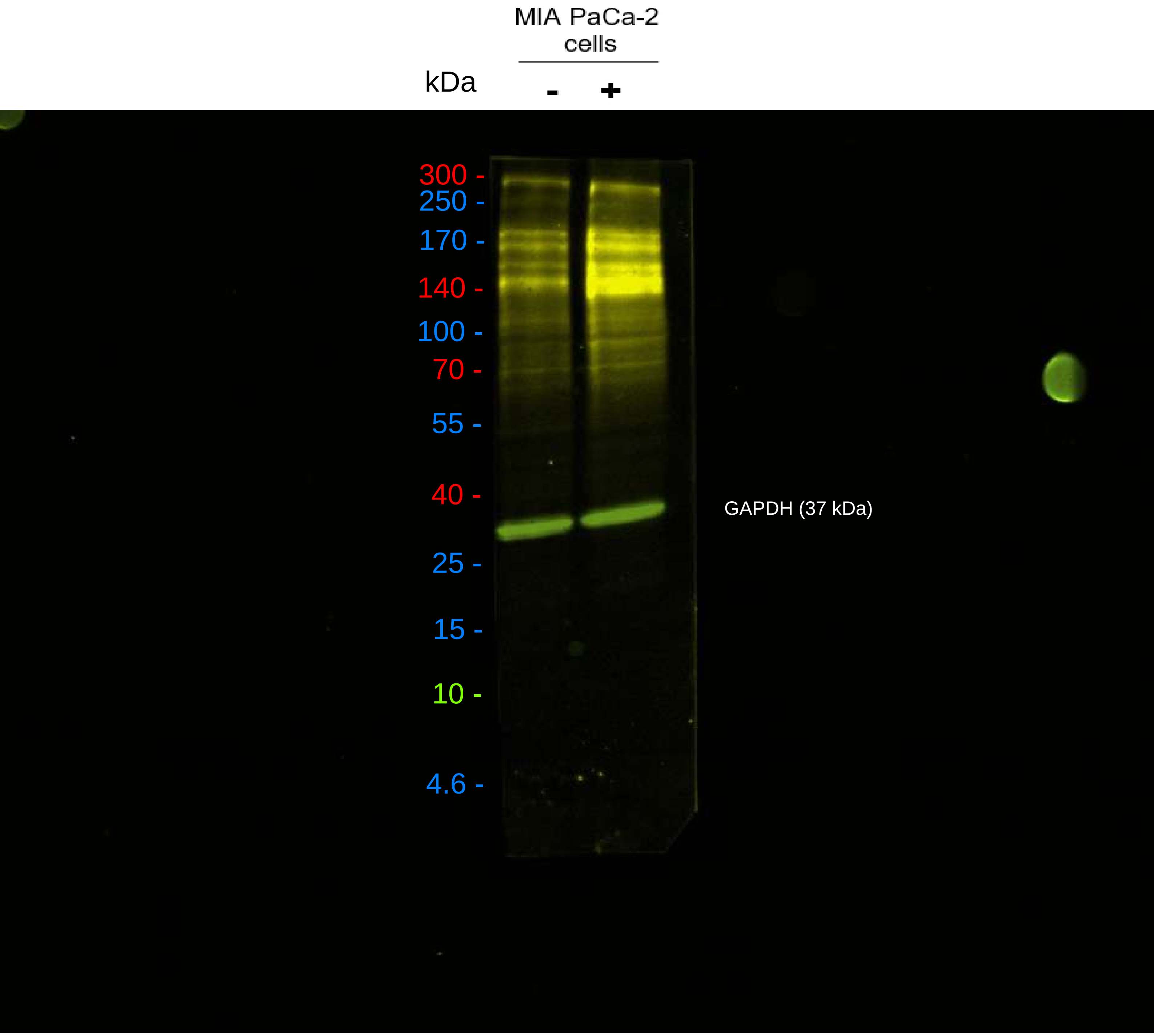

Fig.2B

Western Blot in fluorescence (AF647), time exposure: 202 ms
AAL Lectin (Capan-2)

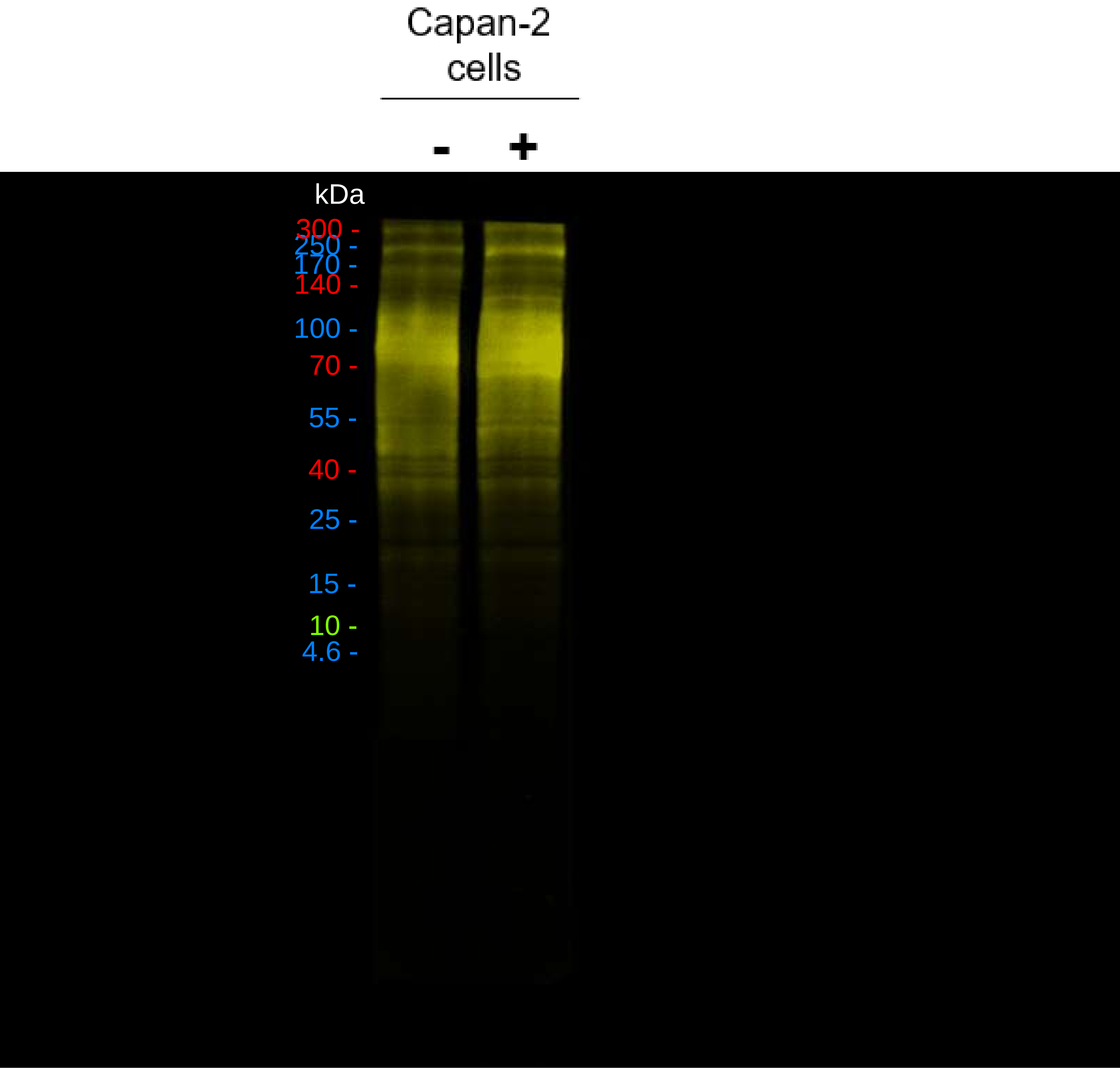

Fig.2B

Western Blot in fluorescence (AF647), time exposure: 1s 56ms
GAPDH (Capan-2)

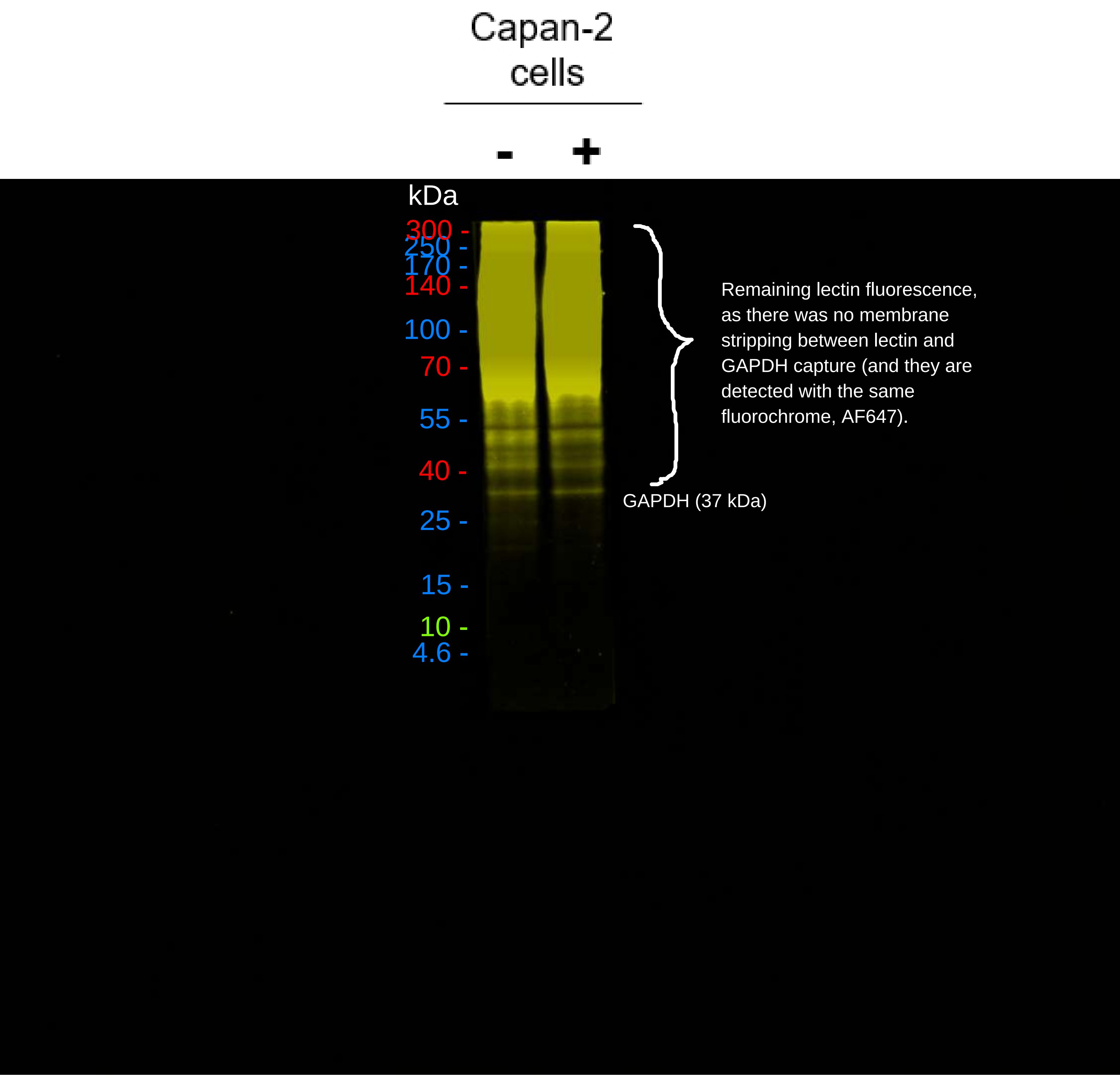

Fig.2B

Western Blot in fluorescence (AF647), time exposure: 859ms
ConA Lectin (PANC-1)

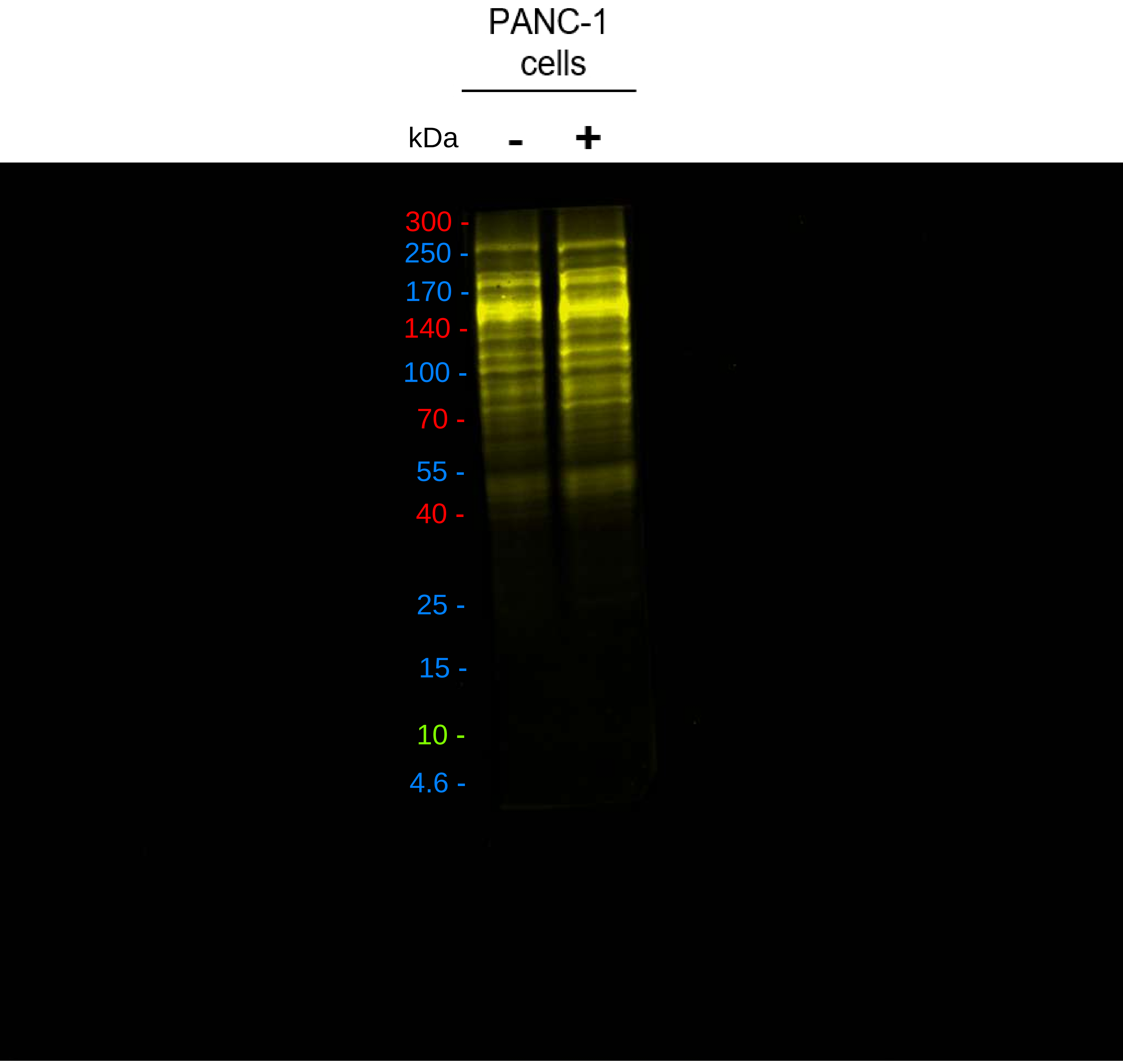

Fig.2B

Western Blot in fluorescence (AF647), exposure time: 1s
GAPDH (PANC-1)

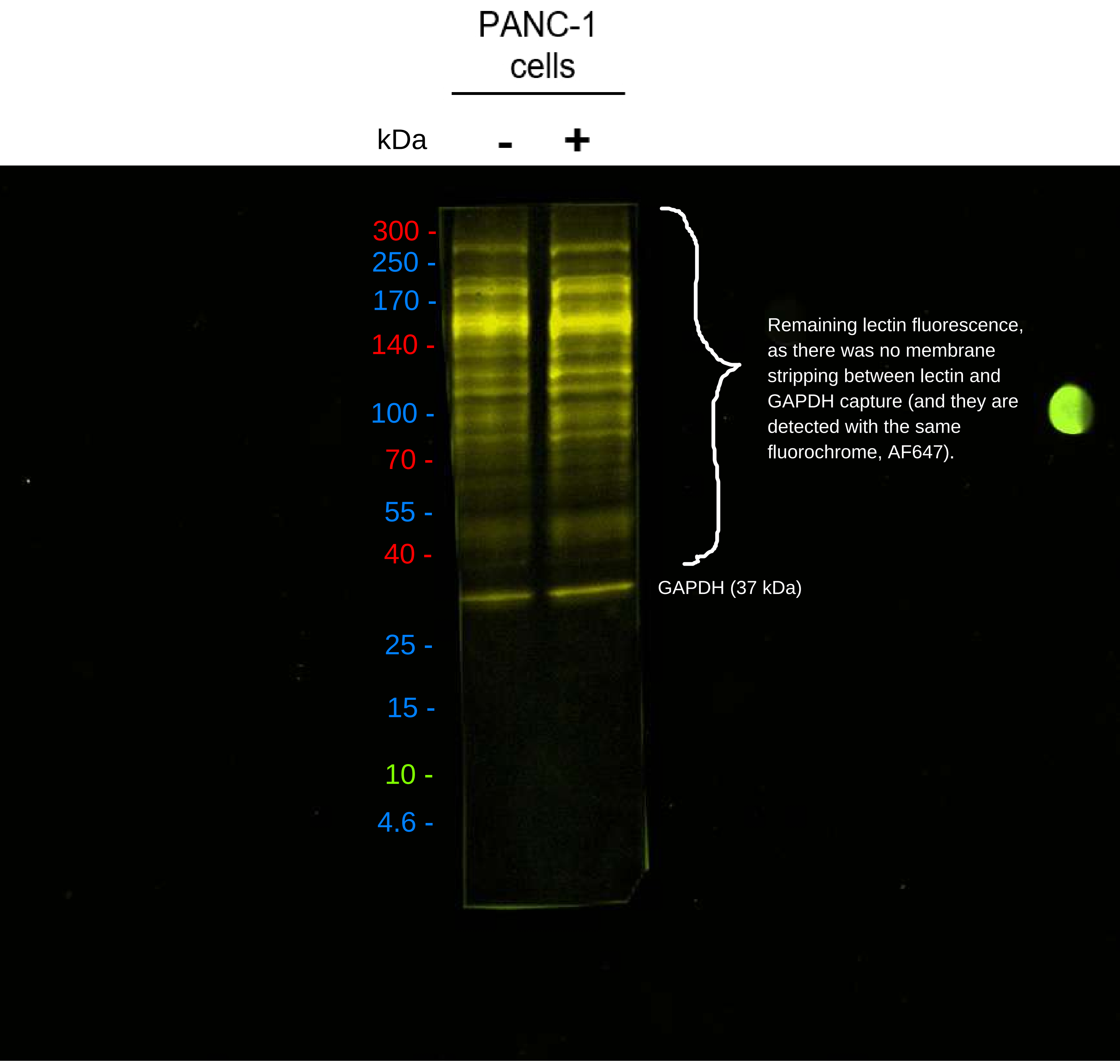

Fig.2B

Western Blot in fluorescence (AF647), time exposure: 1s 582 ms
ConA Lectin (MIA PaCa-2)

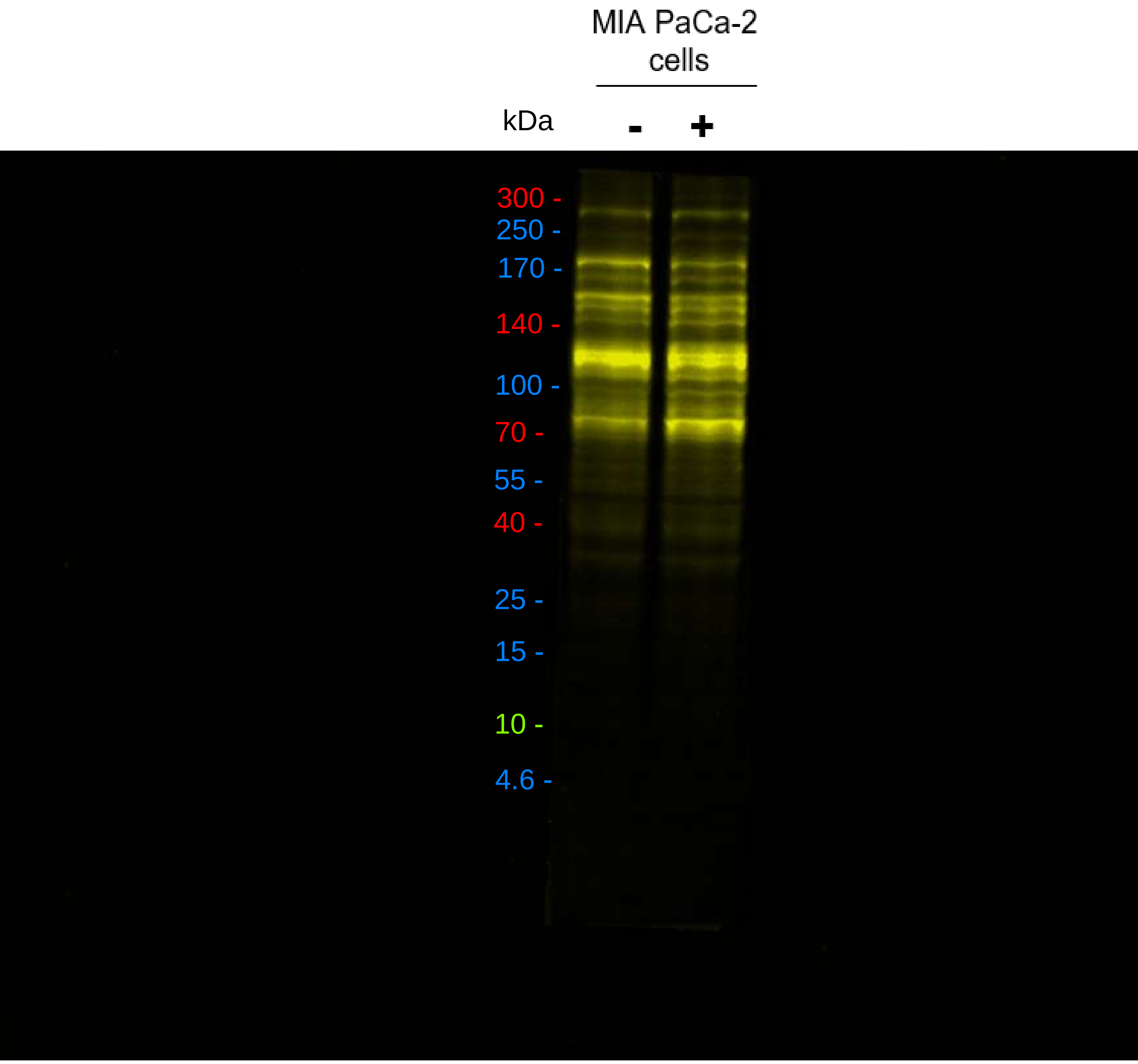

Fig.2B

Western Blot in fluorescence (AF488), multiexposure
GAPDH (MIA PaCa-2)

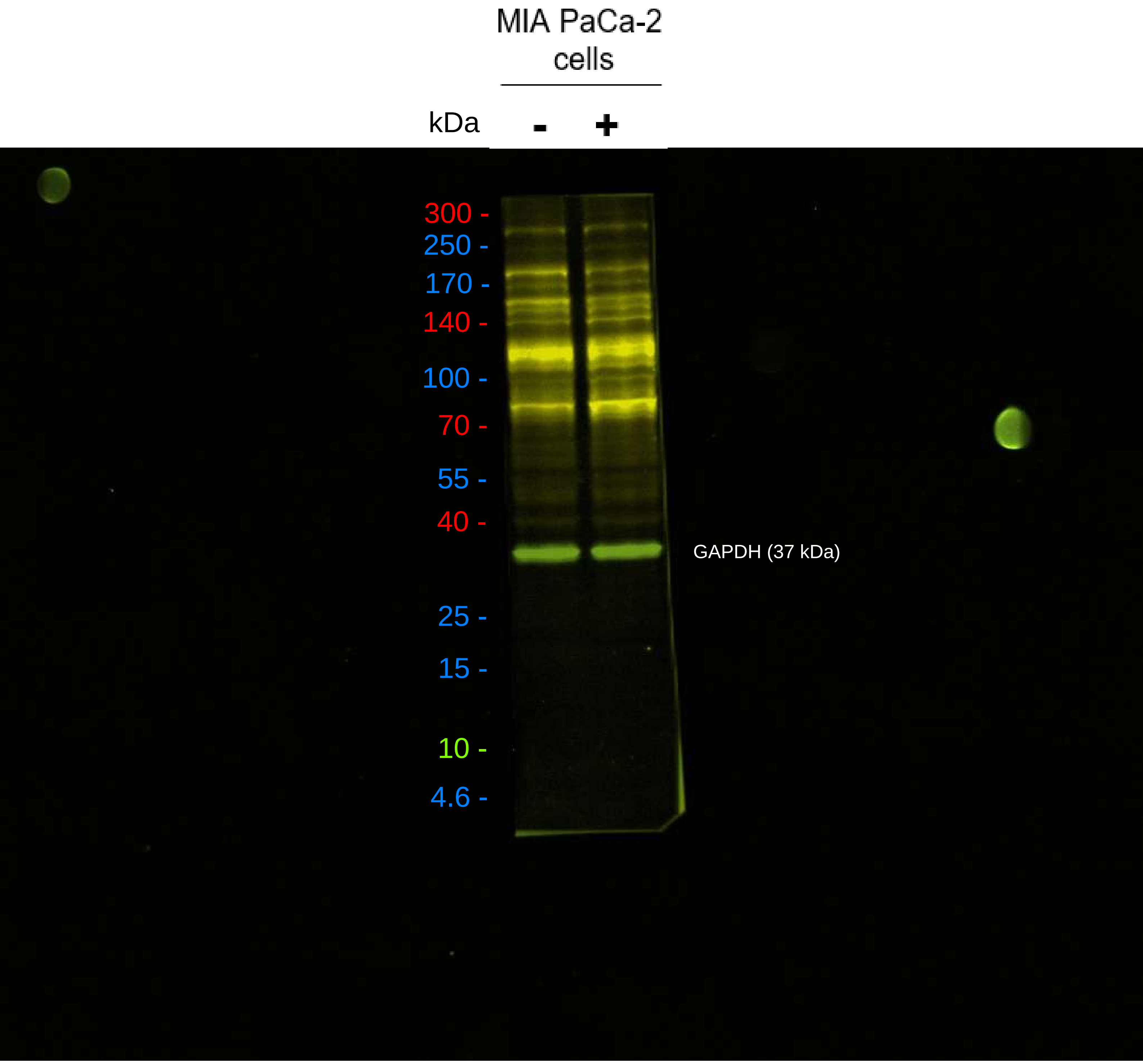

Fig.2B

Western Blot in fluorescence (AF647), exposure time: 773 ms
ConA Lectin (Capan-2)

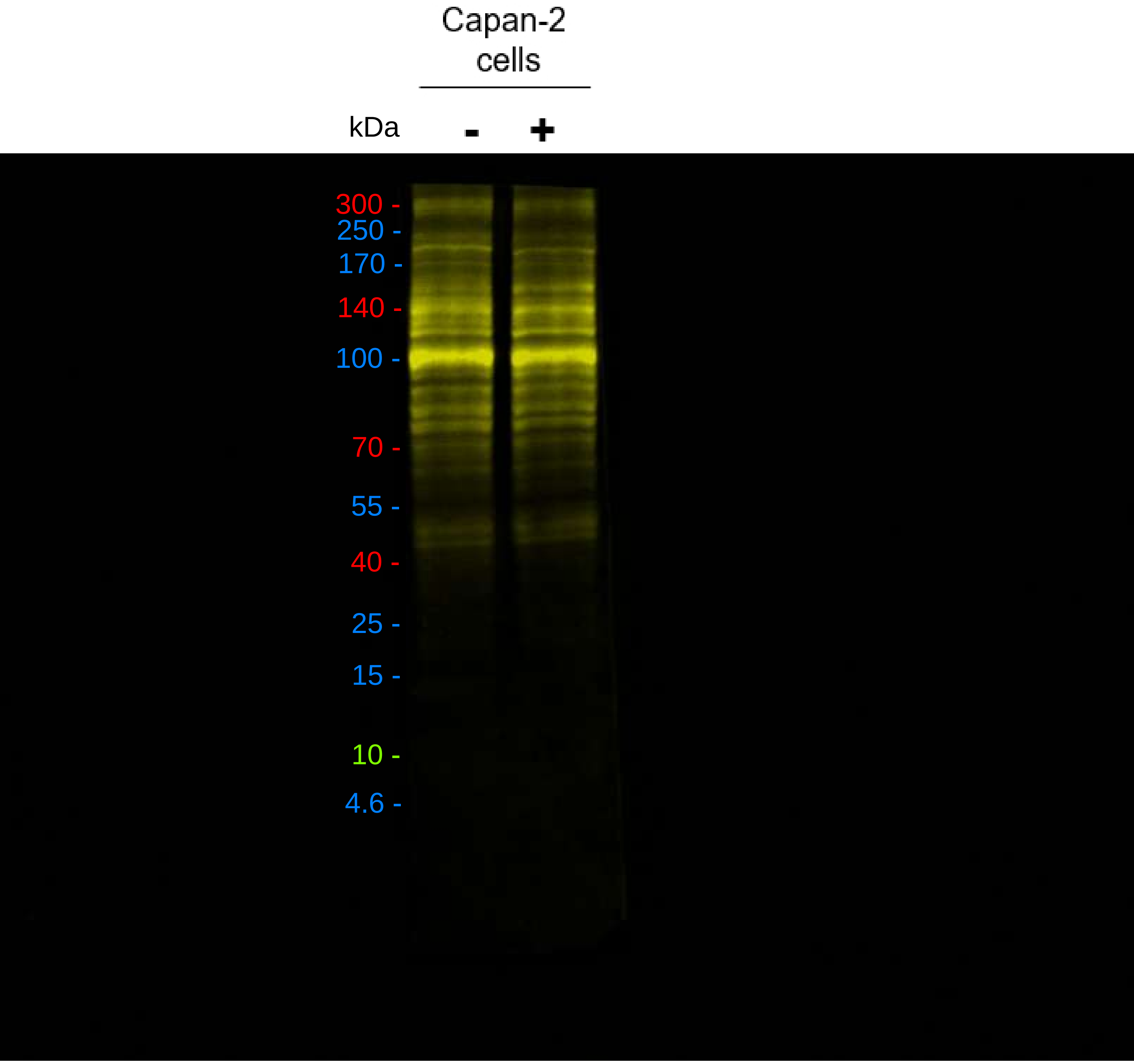

Fig.2B

Western Blot in fluorescence (AF647), exposure time: 1s 260ms
GAPDH (Capan-2)

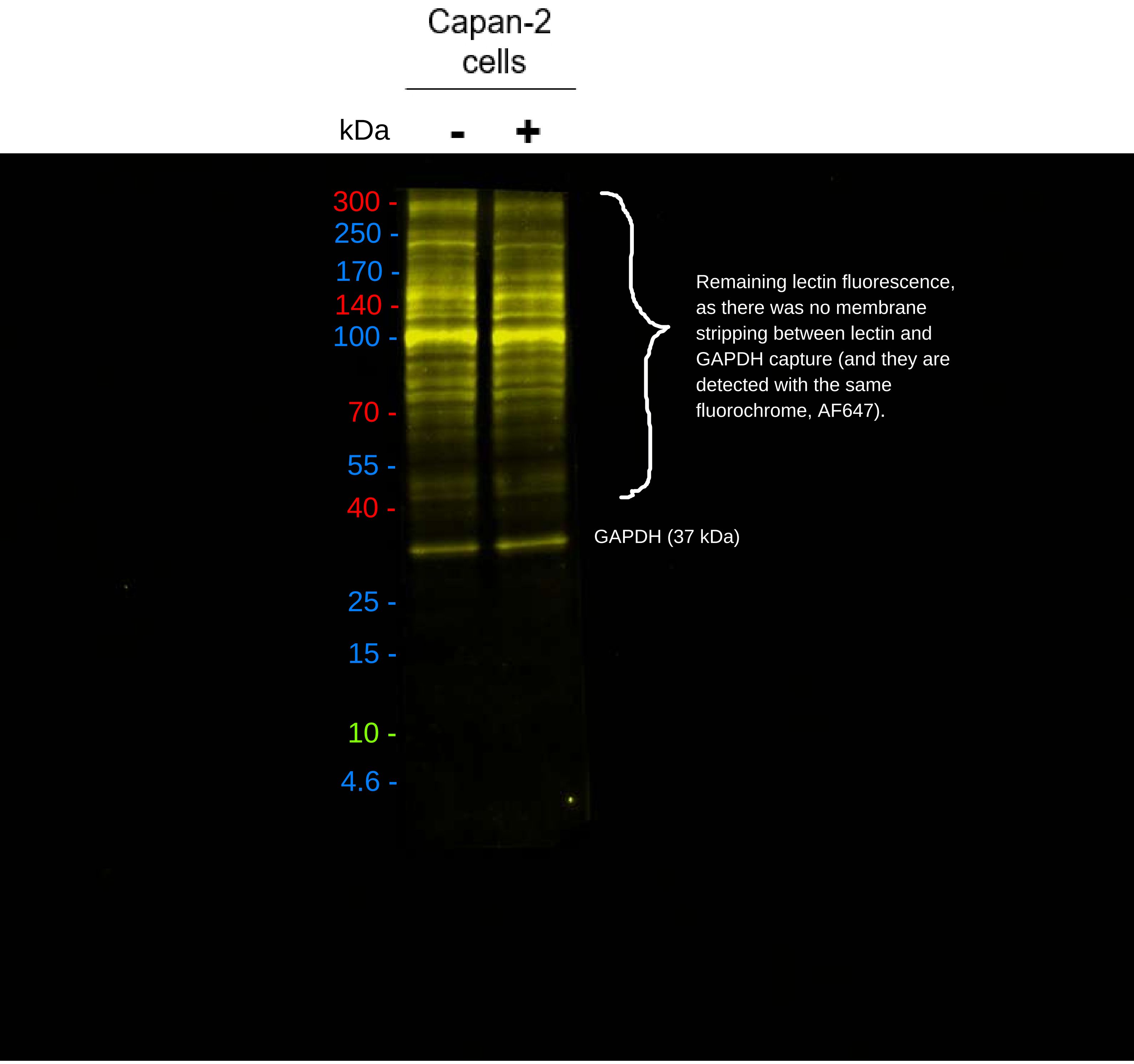

Fig.2B

Western Blot in fluorescence (AF647), exposure time: 587ms
PHA-E Lectin (PANC-1)

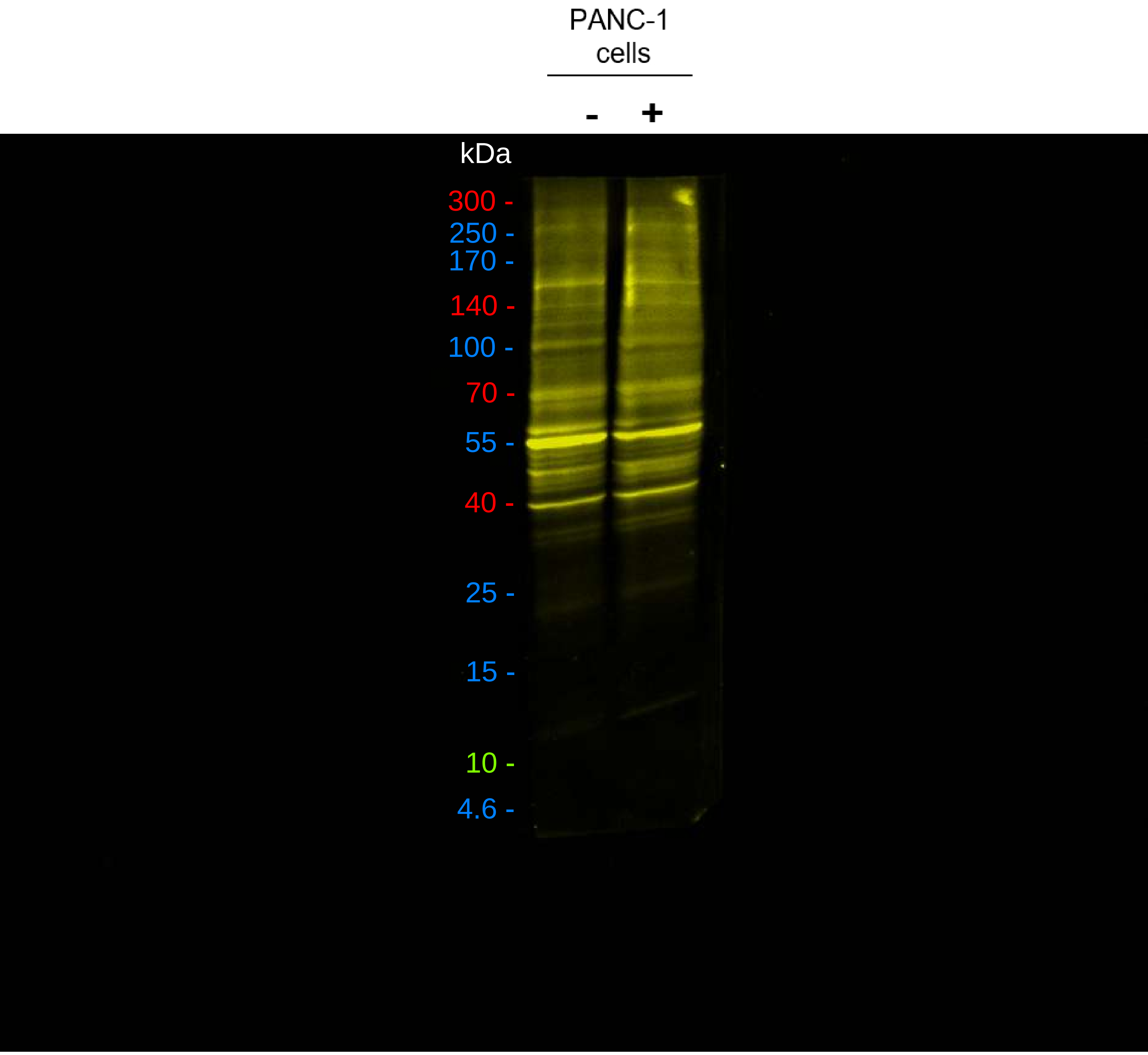

Fig.2B

Western Blot in fluorescence (AF647), exposure time: 902ms
GAPDH (PANC-1)

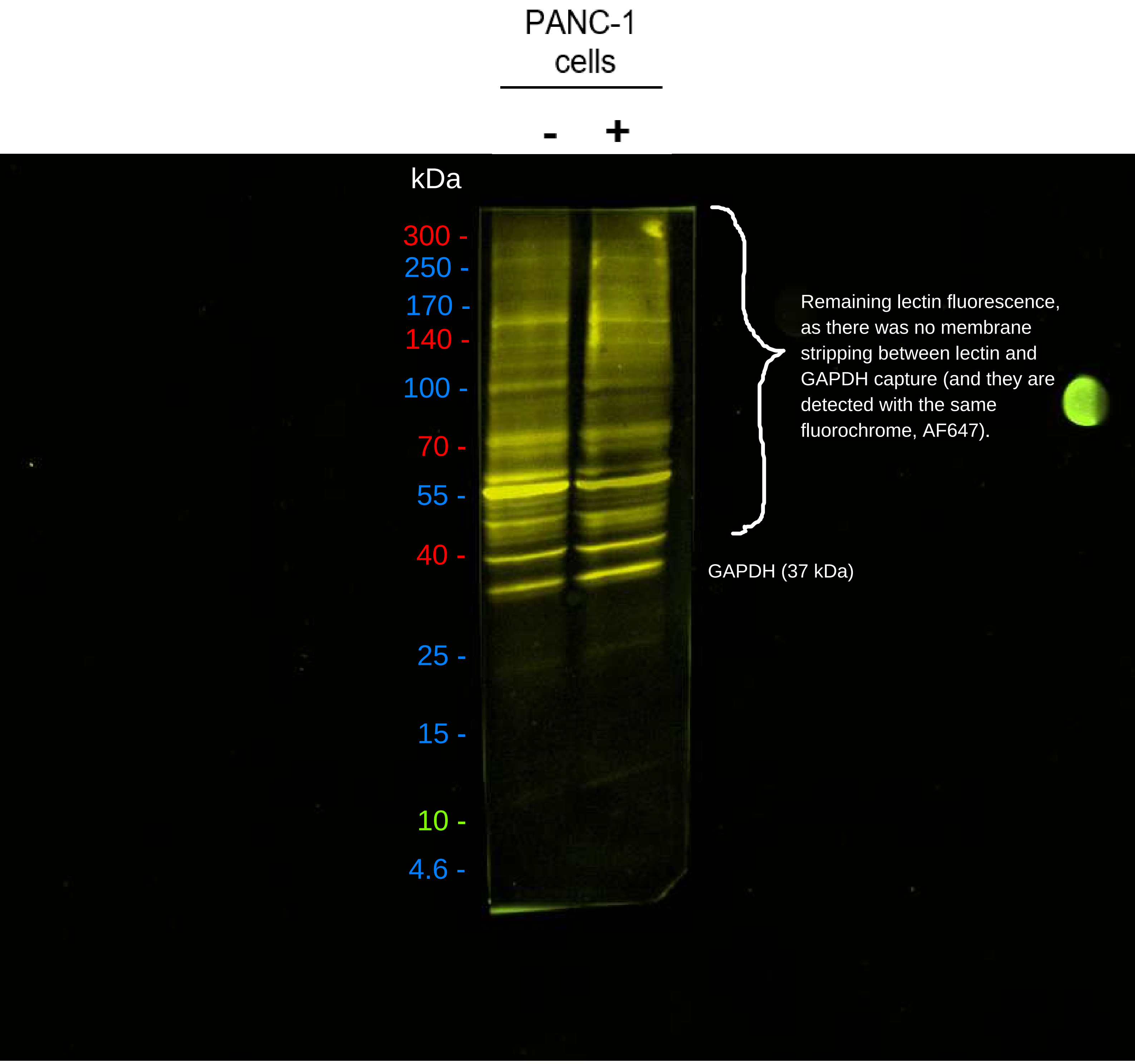

Fig.2B

Western Blot in fluorescence (AF647), exposure time: 883ms
PHA-E Lectin (MIA PaCa-2)

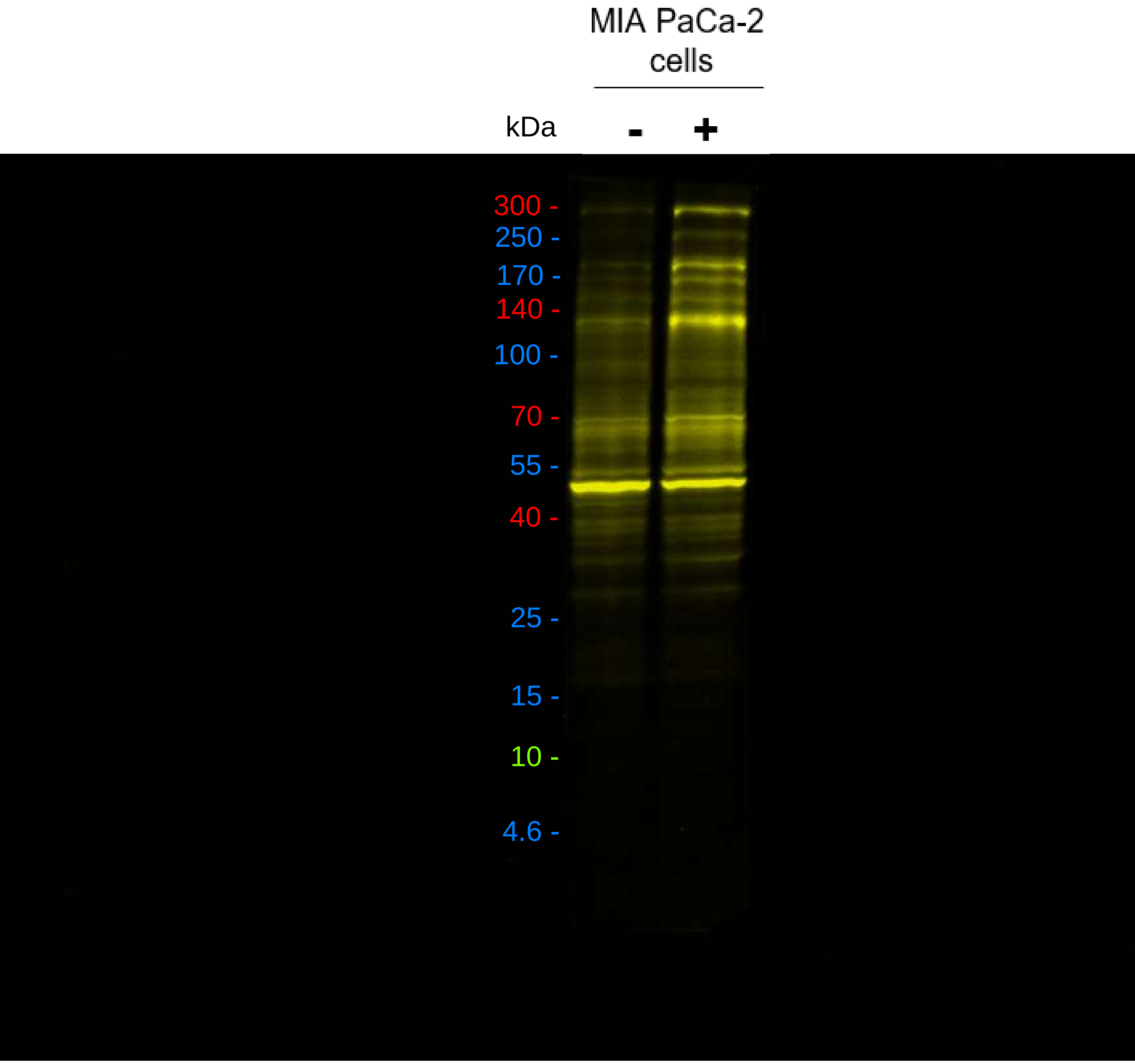

Fig.2B

Western Blot in fluorescence (AF488), multiexposure
GAPDH (MIA PaCa-2)

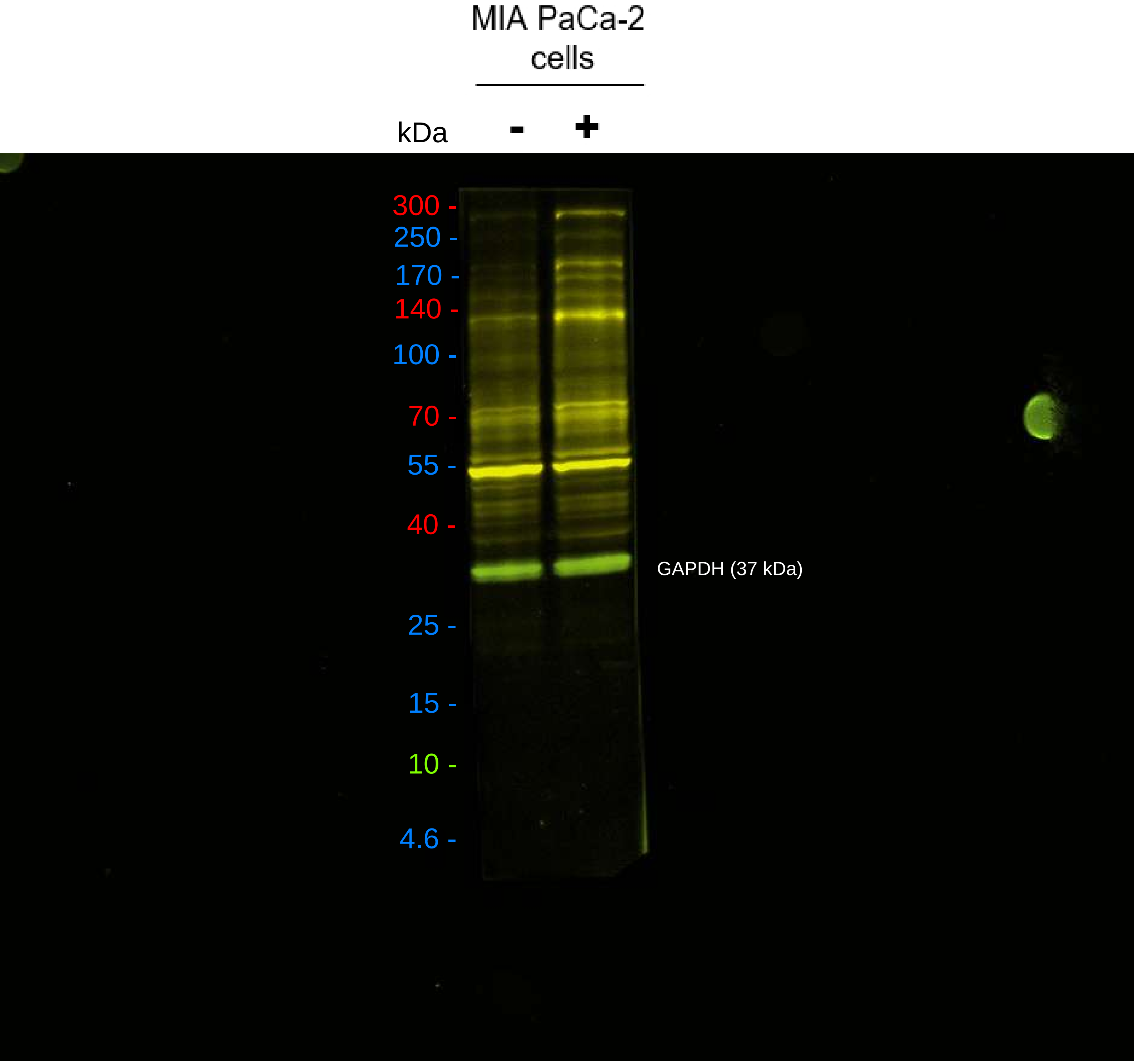

Fig.2B

Western Blot in fluorescence (AF647), time exposure: 283ms
PHA-E Lectin (Capan-2)

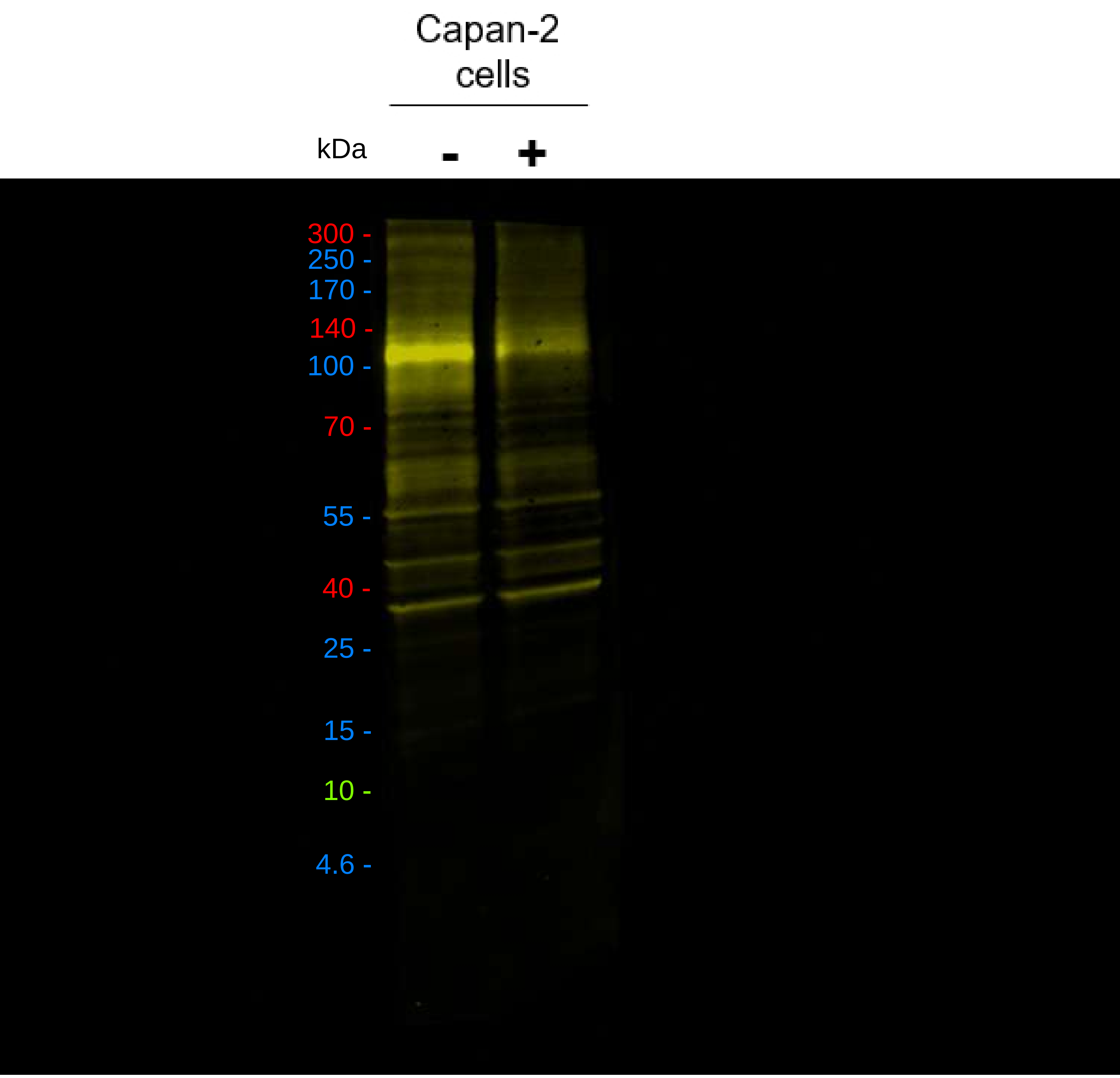

Fig.2B

Western Blot in fluorescence (AF647), time exposure: 334ms
GAPDH (Capan-2)

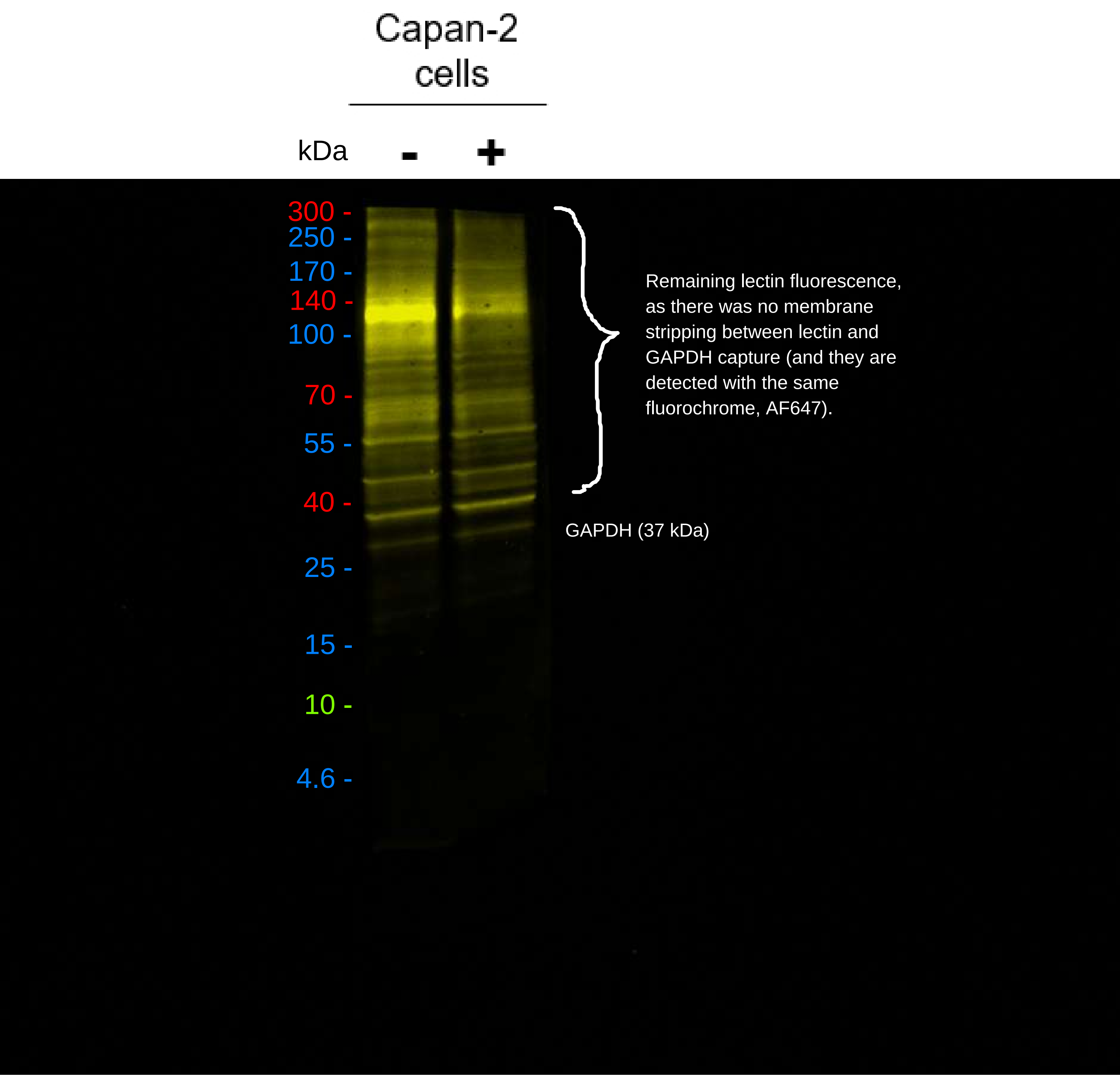

Fig.2E

Western Blot in fluorescence (AF647), time exposure: 1s 382ms
FN1 (PANCO2)

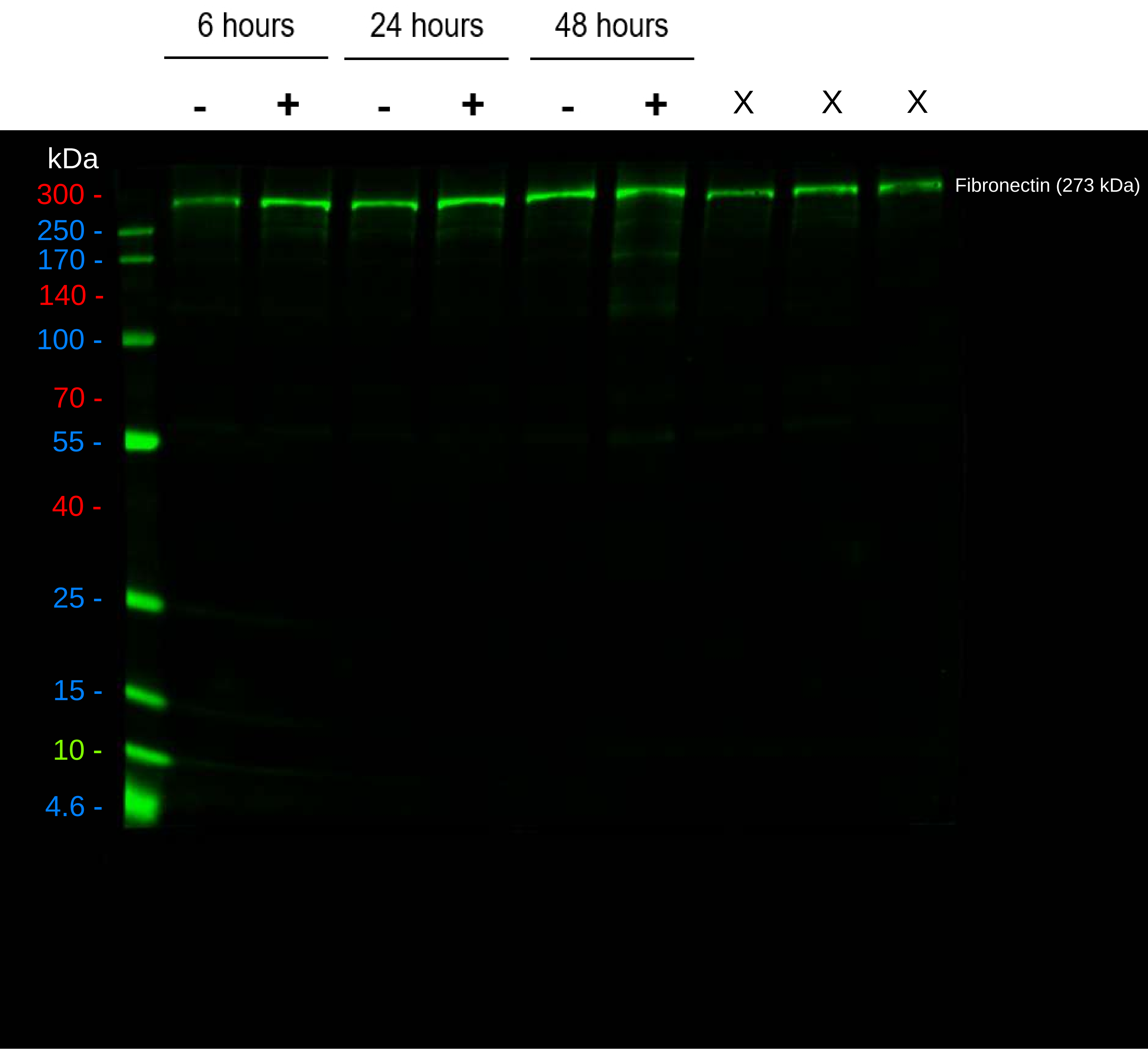

Fig.2E

Western Blot in fluorescence (AF647), time exposure: 8s 873ms
GAPDH (PANCO2)

Remaining FN1 fluorescence,as there was no membrane stripping between FN1 and GAPDH capture (and they are detected with the samefluorochrome, AF647).

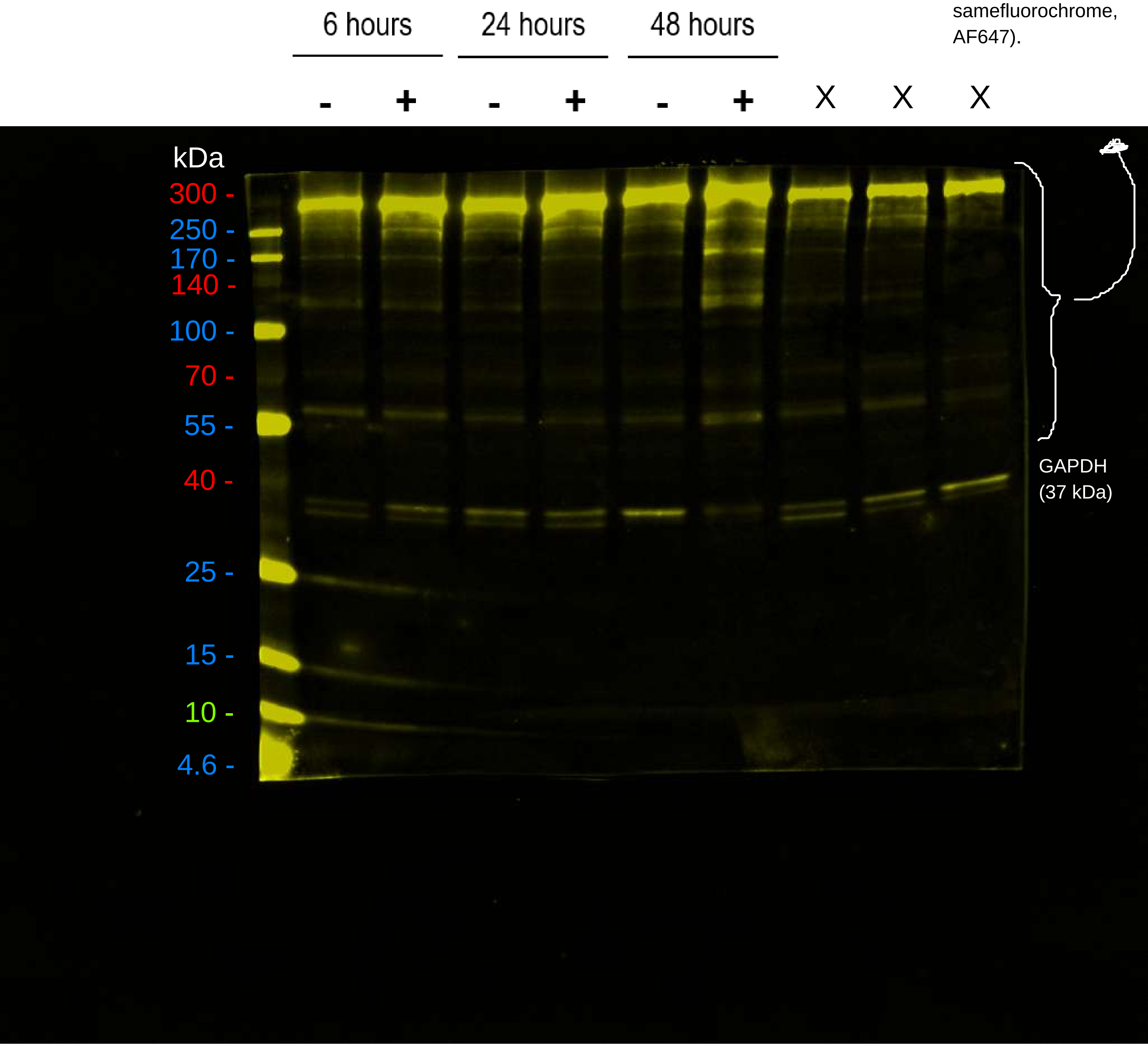

Fig.4E

Western Blot in fluorescence (AF647), time exposure: 5s
FN1 (PANCO2 low FN1)

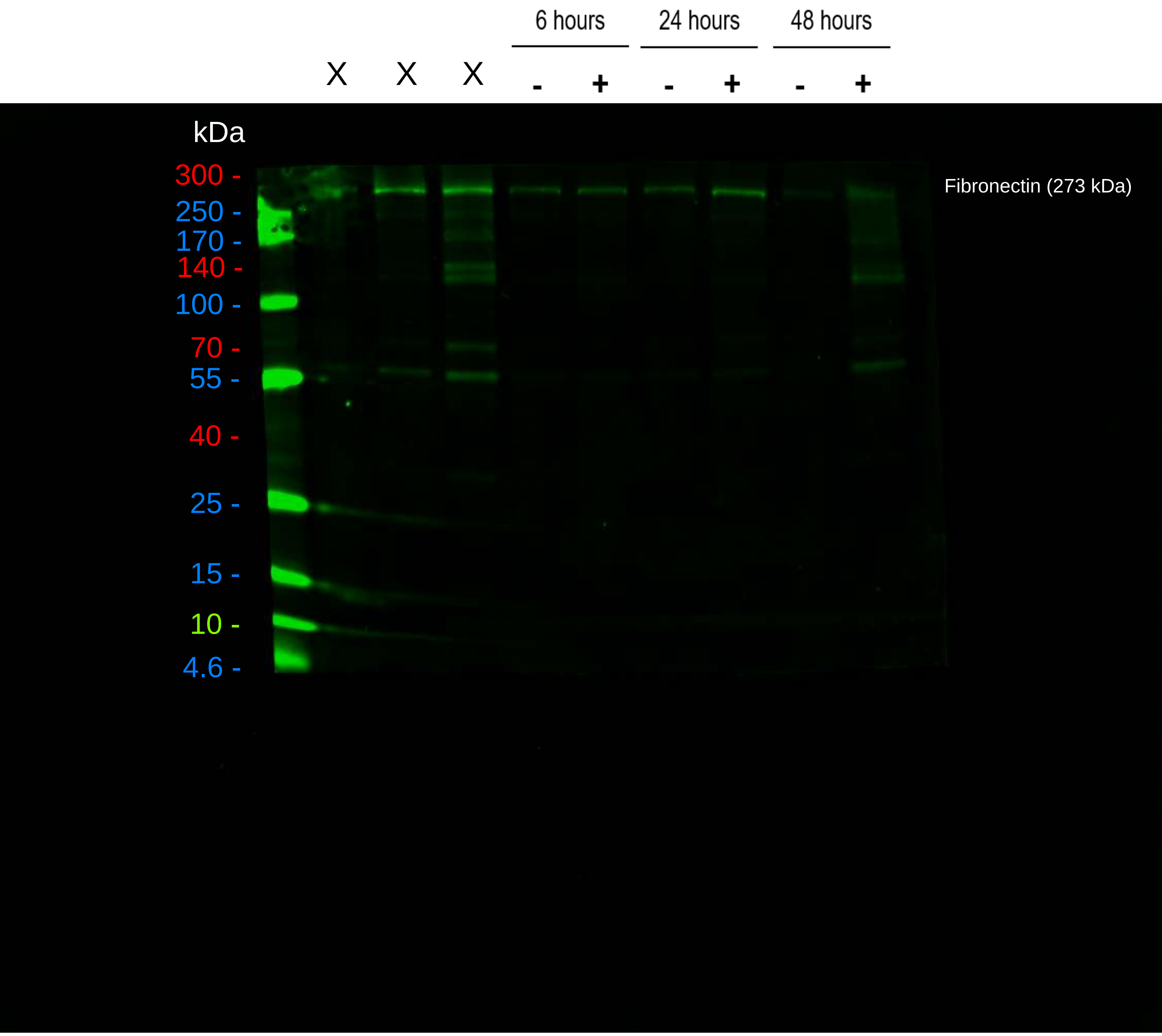

Fig.4E

Western Blot in fluorescence (AF647), time exposure: 6s 721ms
GAPDH (PANCO2 low FN1)

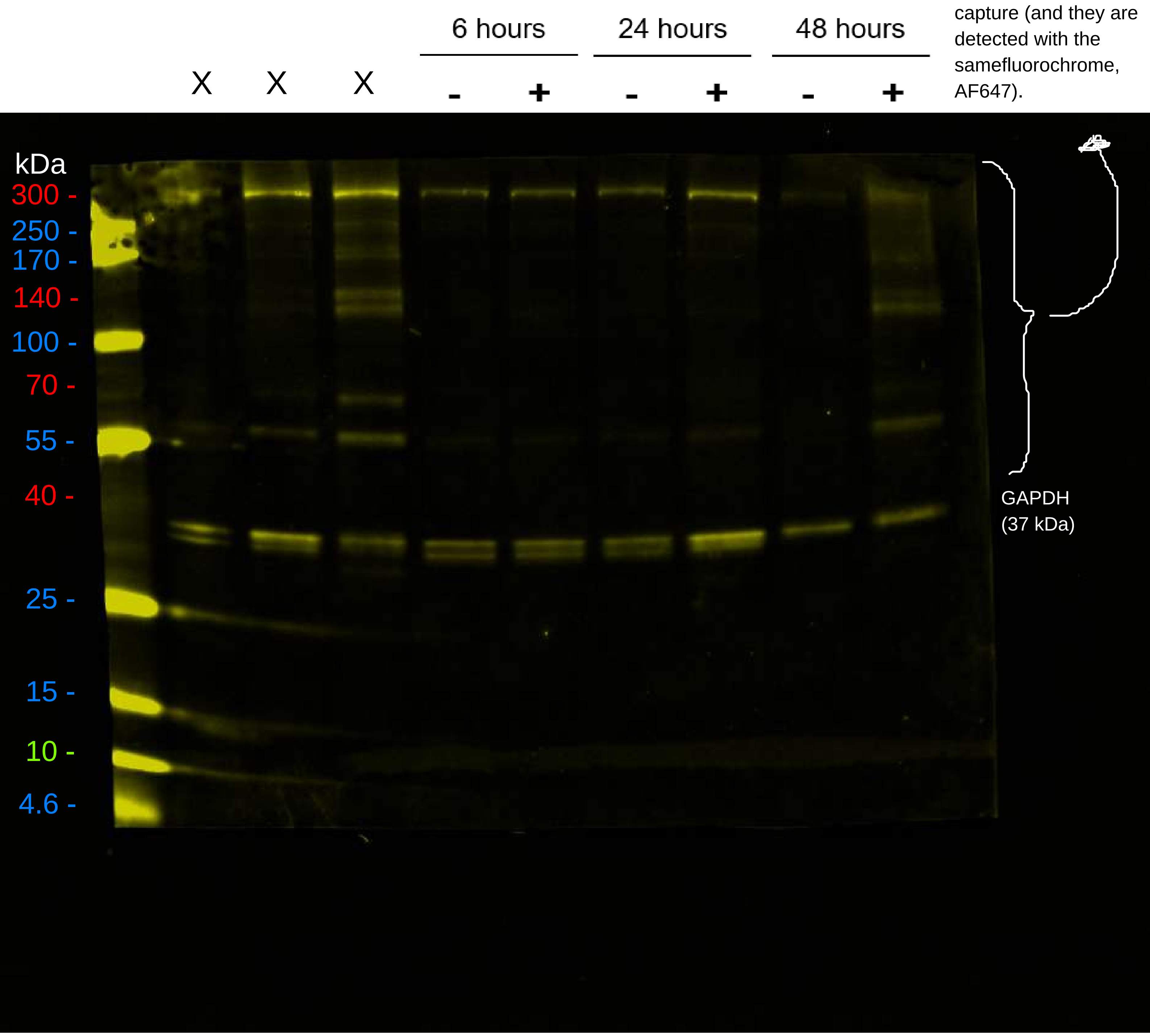

Fig.S2

Western Blot in fluorescence (FITC), time exposure: 255ms
PHA-L Lectin (PANC-1)

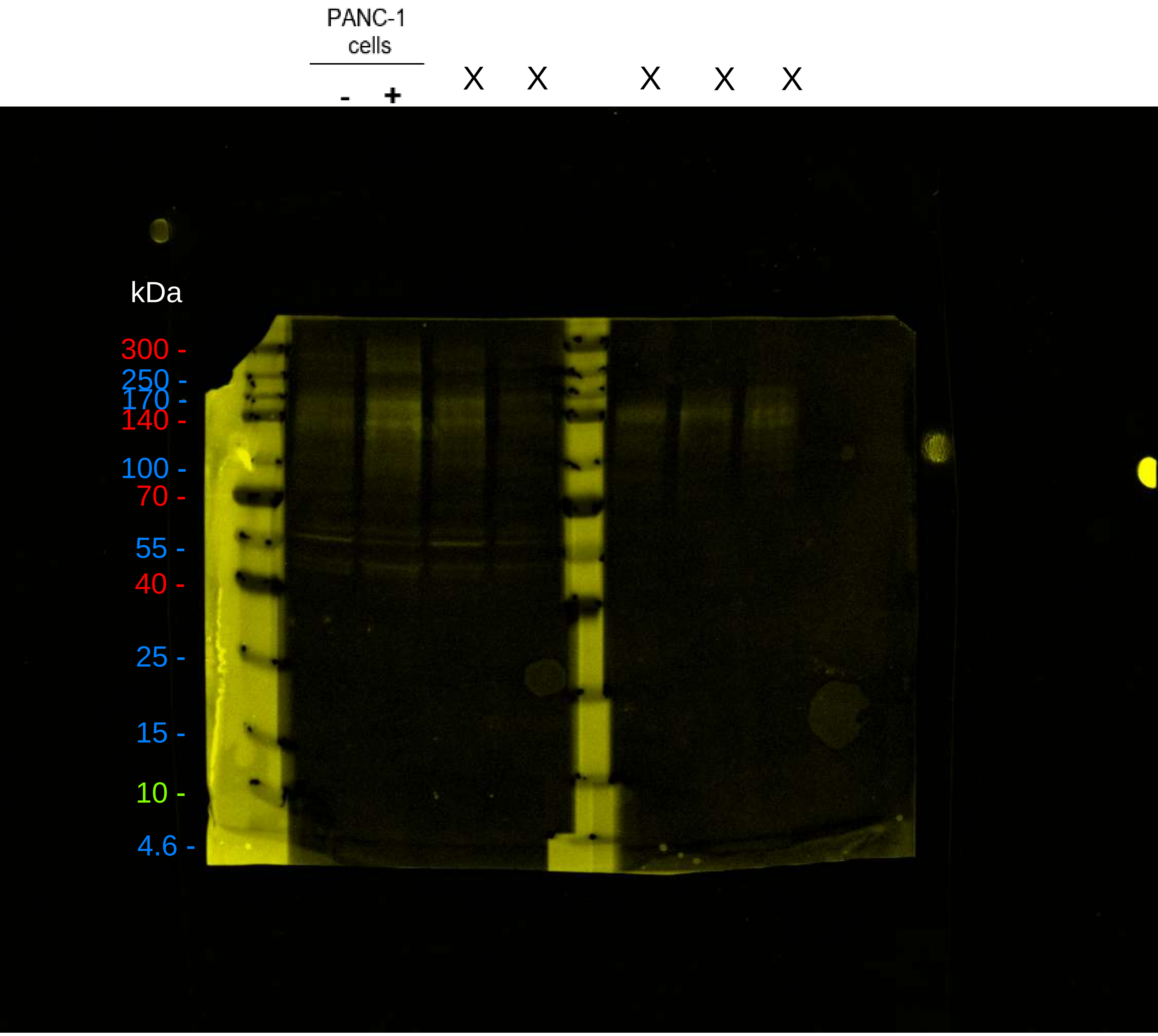

Fig.S2

Western Blot in fluorescence (AF647), time exposure: 951ms
GAPDH (PANC-1)

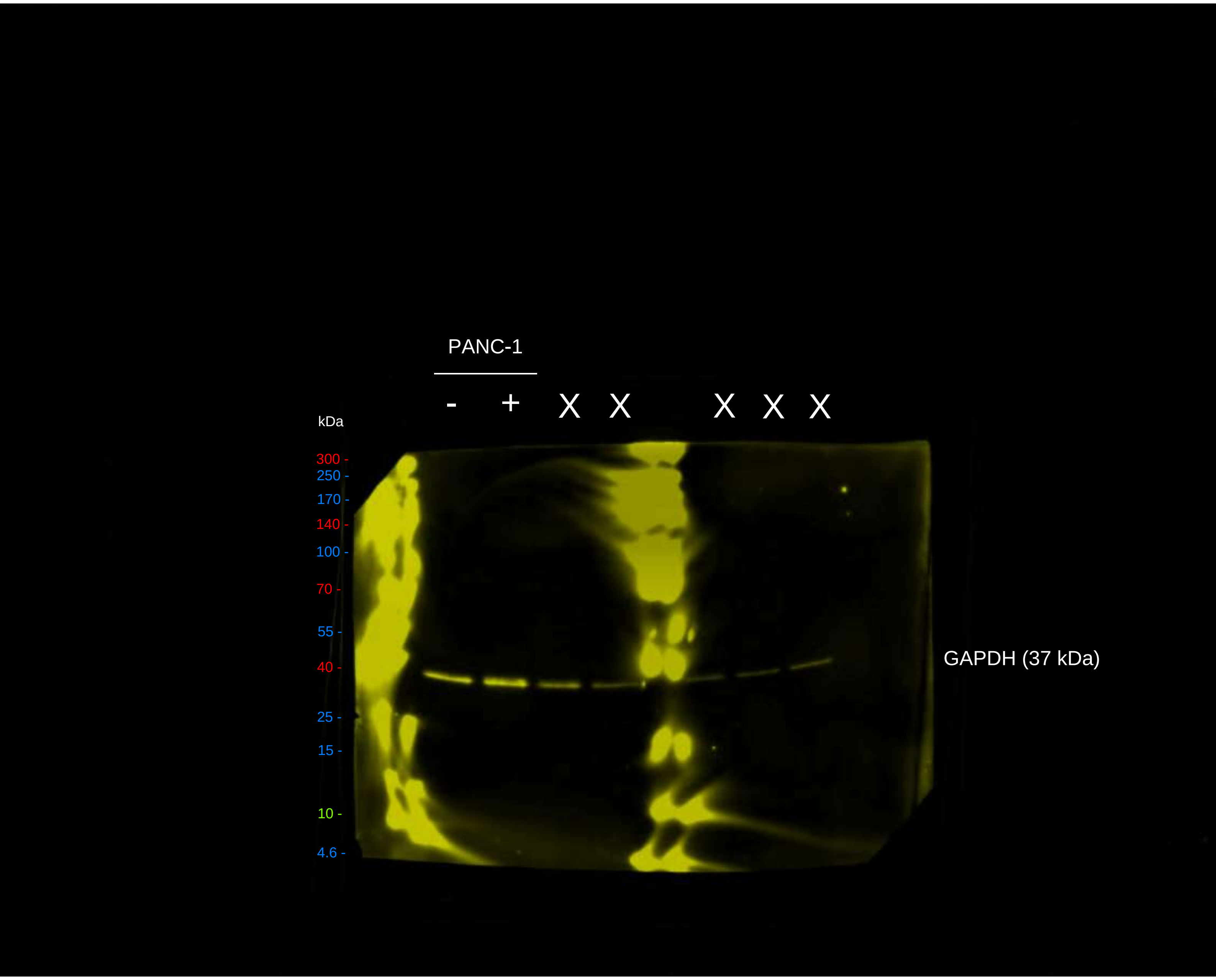

Fig.S3

Western Blot in fluorescence (AF647), time exposure: 2s 691ms
MGAT2 (PANC-1)

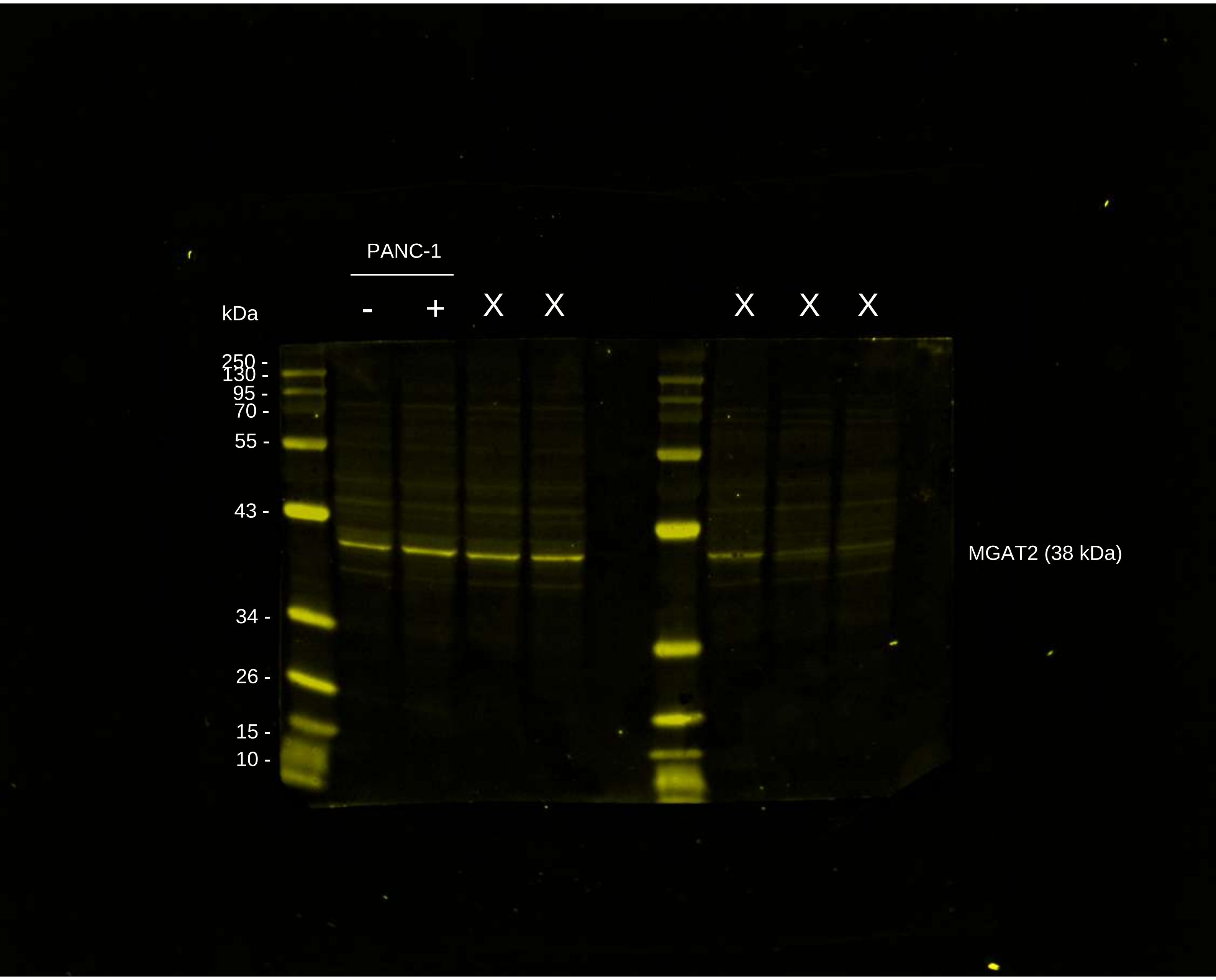

Fig.S3

Western Blot in fluorescence (AF647), time exposure: 1s 490ms
GAPDH (PANC-1)

Remaining MGAT2 fluorescence, as there was no membrane stripping between MGAT2 and GAPDH capture (and they are detected with the same fluorochrome, AF647).

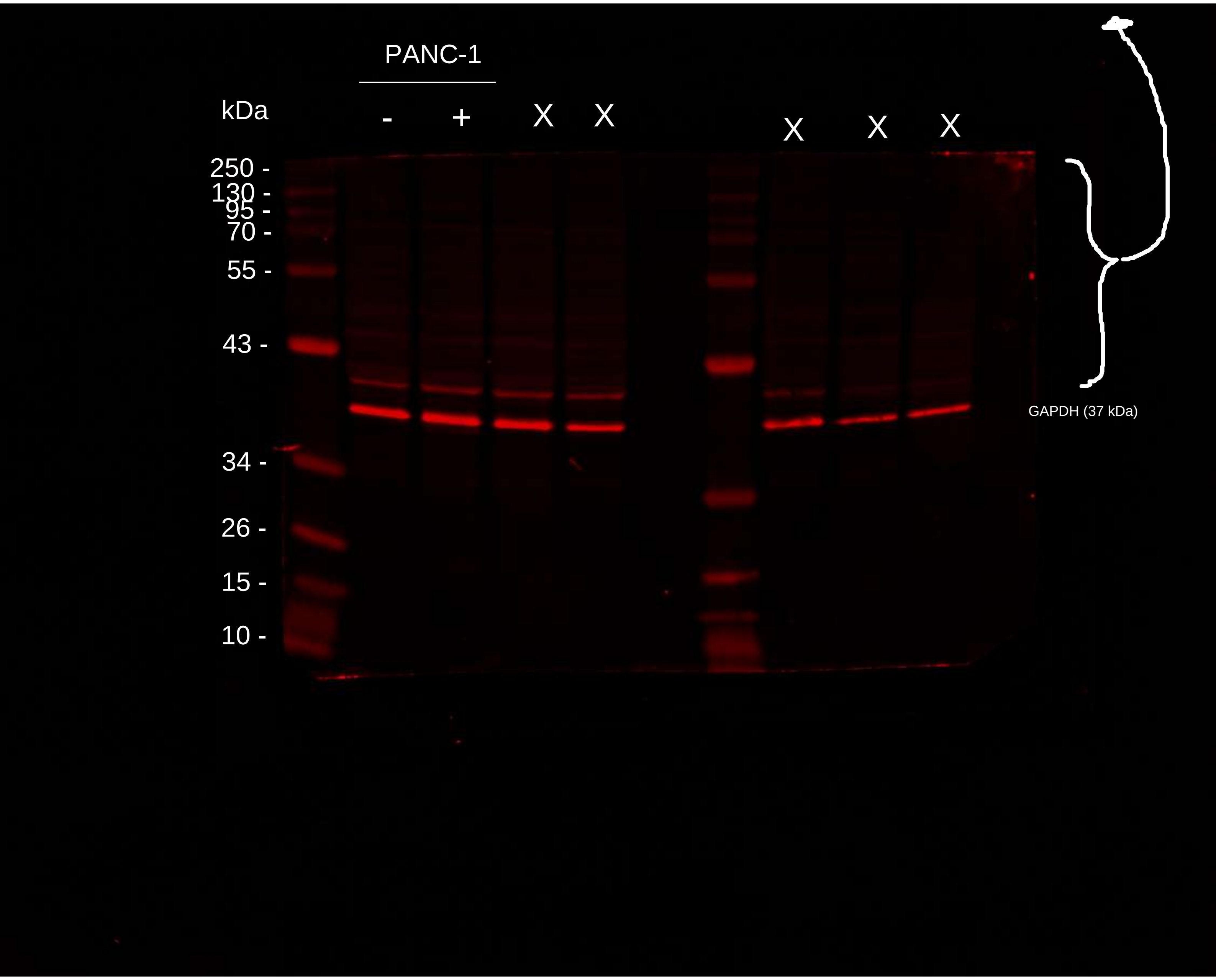

Supplement: S1 File — (PDF) [file pone.0317096.s004.pdf]
